# A systems genetics resource and analysis of sleep regulation in the mouse

**DOI:** 10.1371/journal.pbio.2005750

**Published:** 2018-08-09

**Authors:** Shanaz Diessler, Maxime Jan, Yann Emmenegger, Nicolas Guex, Benita Middleton, Debra J. Skene, Mark Ibberson, Frederic Burdet, Lou Götz, Marco Pagni, Martial Sankar, Robin Liechti, Charlotte N. Hor, Ioannis Xenarios, Paul Franken

**Affiliations:** 1 Center for Integrative Genomics, University of Lausanne, Switzerland; 2 Vital-IT Systems Biology Division, SIB Swiss Institute of Bioinformatics, Lausanne, Switzerland; 3 Faculty of Health and Medical Sciences, University of Surrey, Guildford, United Kingdom; Charité Universitätsmedizin Berlin, Germany

## Abstract

Sleep is essential for optimal brain functioning and health, but the biological substrates through which sleep delivers these beneficial effects remain largely unknown. We used a systems genetics approach in the BXD genetic reference population (GRP) of mice and assembled a comprehensive experimental knowledge base comprising a deep “sleep-wake” phenome, central and peripheral transcriptomes, and plasma metabolome data, collected under undisturbed baseline conditions and after sleep deprivation (SD). We present analytical tools to interactively interrogate the database, visualize the molecular networks altered by sleep loss, and prioritize candidate genes. We found that a one-time, short disruption of sleep already extensively reshaped the systems genetics landscape by altering 60%–78% of the transcriptomes and the metabolome, with numerous genetic loci affecting the magnitude and direction of change. Systems genetics integrative analyses drawing on all levels of organization imply α-amino-3-hydroxy-5-methyl-4-isoxazolepropionic acid (AMPA) receptor trafficking and fatty acid turnover as substrates of the negative effects of insufficient sleep. Our analyses demonstrate that genetic heterogeneity and the effects of insufficient sleep itself on the transcriptome and metabolome are far more widespread than previously reported.

## Author summary

Sleep is essential for optimal brain functioning and health, but the biological substrates through which sleep delivers these beneficial effects remain largely unknown. We used a systems genetics approach in a large, diverse reference population of mice and assembled a comprehensive experimental knowledge base comprising “sleep-wake” data, central and peripheral gene expression, and plasma metabolic indicators, collected under undisturbed baseline conditions and after sleep deprivation (SD). We present analytical tools to interactively interrogate the database, visualize the molecular networks altered by sleep loss, and prioritize candidate genes. We found that a brief, one-time disruption of sleep extensively reshaped the transcriptome in cerebral cortex and liver, and the plasma metabolome, with numerous genetic loci affecting the magnitude and direction of change. Integrative analyses drawing on multiple sources of data imply α-amino-3-hydroxy-5-methyl-4-isoxazolepropionic acid (AMPA) receptor trafficking and fatty acid turnover as substrates of the negative effects of insufficient sleep. Our analyses demonstrate that genetic heterogeneity and the effects of insufficient sleep on gene expression and metabolism are far more widespread than previously reported.

## Introduction

Insufficient or disrupted sleep characterizes the 24 h lifestyle of modern society and represents a serious public health concern, as it is associated with increased risk for, e.g., obesity, diabetes, and high blood pressure, and impairs cognitive performance, which in turn increases the likelihood of accidents, medical errors, and loss of productivity [1,2]. Several hypotheses concerning sleep’s still elusive function converge on the notion that staying awake imposes a burden that can only be efficiently alleviated during sleep [3–7]. This concept of a need for sleep accumulating during wakefulness and recovering while asleep is central in sleep research and is referred to as sleep homeostasis. Insight into the molecular substrates of the sleep homeostatic process is instrumental in advancing our basic understanding of sleep need under both physiological and pathological conditions.

The impact of acute sleep deprivation (SD) on recovery sleep and cognitive performance is under strong genetic control [8–13], and genetic approaches therefore seem promising in uncovering the molecular pathways important in sleep homeostasis. Reductionist studies in mice and flies deleting genes through gene targeting (for review, see [8]) or in mutagenesis screens [14–16] have demonstrated that single genes can have large effects on various aspects of sleep, including its homeostatic regulation. Such large single-gene (mendelian) effects—often assessed on 1 genetic background only—are, however, likely to be the exception. Indeed, susceptibility to sleep loss in the general population is assumed to be determined by the interactions of many genes, their natural allelic variants, and their interaction with the environment (lifestyle), a complexity that only recently has begun to be appreciated. Such complexity can best be assessed in so-called genetic reference populations (GRPs), which are designed for the study of complex traits inherited in a nonmendelian fashion. The BXD panel of advanced recombinant inbred lines (ARILs) is the largest and best-characterized GRP to date, consisting of well over 150 lines in which 2 parental (C57BL/6J [B6] and DBA/2J [D2]), now fully sequenced genomes are segregating (www.genenetwork.org; [17]). As each line represents a reproducible clone of animals, many mutually reinforcing datasets can be collected and compared at multiple levels across many biological systems. This approach has been termed “systems genetics,” which in essence allows for making inferences about biological phenomena by assessing the flow of information from DNA to phenotype at the level of a population and how this flow is perturbed by environmental challenges. Because systems genetics generalizes results to a population level, it is considered critical for predicting disease susceptibility [18]. Systems genetics has been applied with great success in the BXD set for, e.g., mitochondrial function and metabolic- and aging-related phenotypes [19–21].

Systems genetics approaches for sleep have been pioneered in the fly and mouse [22,23], but neither study reported on the effects of sleep loss on intermediate phenotypes, such as the metabolome and transcriptome. Here, we present an extensive and comprehensive dataset interrogating the BXD set at the levels of the genome, the brain and liver transcriptomes, the plasma metabolome, and finally, the phenome including sleep-wake state, electroencephalography (EEG)-, and locomotor activity (LMA)-related phenotypes, both under undisturbed baseline conditions and after an acute SD challenging the sleep homeostatic process. We observed that SD profoundly impacted all 3 phenotypic levels and that genetic background not only determined the magnitude but also the direction of the SD-evoked changes. The molecular pathways associated with these effects will be illustrated here to introduce our integrated data resource. The molecular signaling circuitry underlying the equally profound phenotypic differences observed under baseline conditions will be reported in subsequent molecular-driven validations.

Systems genetics is an emerging field, and innovative ways to improve data access, portability, and reproducibility; tools to display and mine these data; and statistical models to extract the multidimensional relationships across datasets are areas of intense research [24]. The size and complexity of our current dataset necessitated the development of new analytical tools and data sharing strategies such as (i) a supervised machine learning–based algorithm to annotate sleep-wake states on EEG/electromyogram (EMG) tracks, (ii) a gene-prioritization strategy that draws on all levels of the experimental dataset to assist the search for candidate genes within quantitative trait locus (QTL) intervals, and (iii) the implementation and integration of a recently developed systems genetics visualization tool [25] in a dynamic web-based interface that, in addition, provides access to the data presented and enables interactive data mining (https://bxd.vital-it.ch).

## Results

This section is organized as follows: study design and the types of data contributing to our resource are shortly described first. We then ascertain the contribution of genetic factors to all the intermediate and end phenotypes we quantified. Next, the tools to interactively visualize the systems genetics relationships and to prioritize candidate genes will be described in detail. Because our current focus is on the effects of enforced wakefulness, we describe the SD-evoked changes at the level of the GRP, as well as the genetic effects thereon, before closing with 4 examples that, aided with the prioritization tool, point to novel molecular pathways shaping the marked genetic variability in the response to sleep loss at all levels of organization.

### Study design and input data

**Fig 1:**
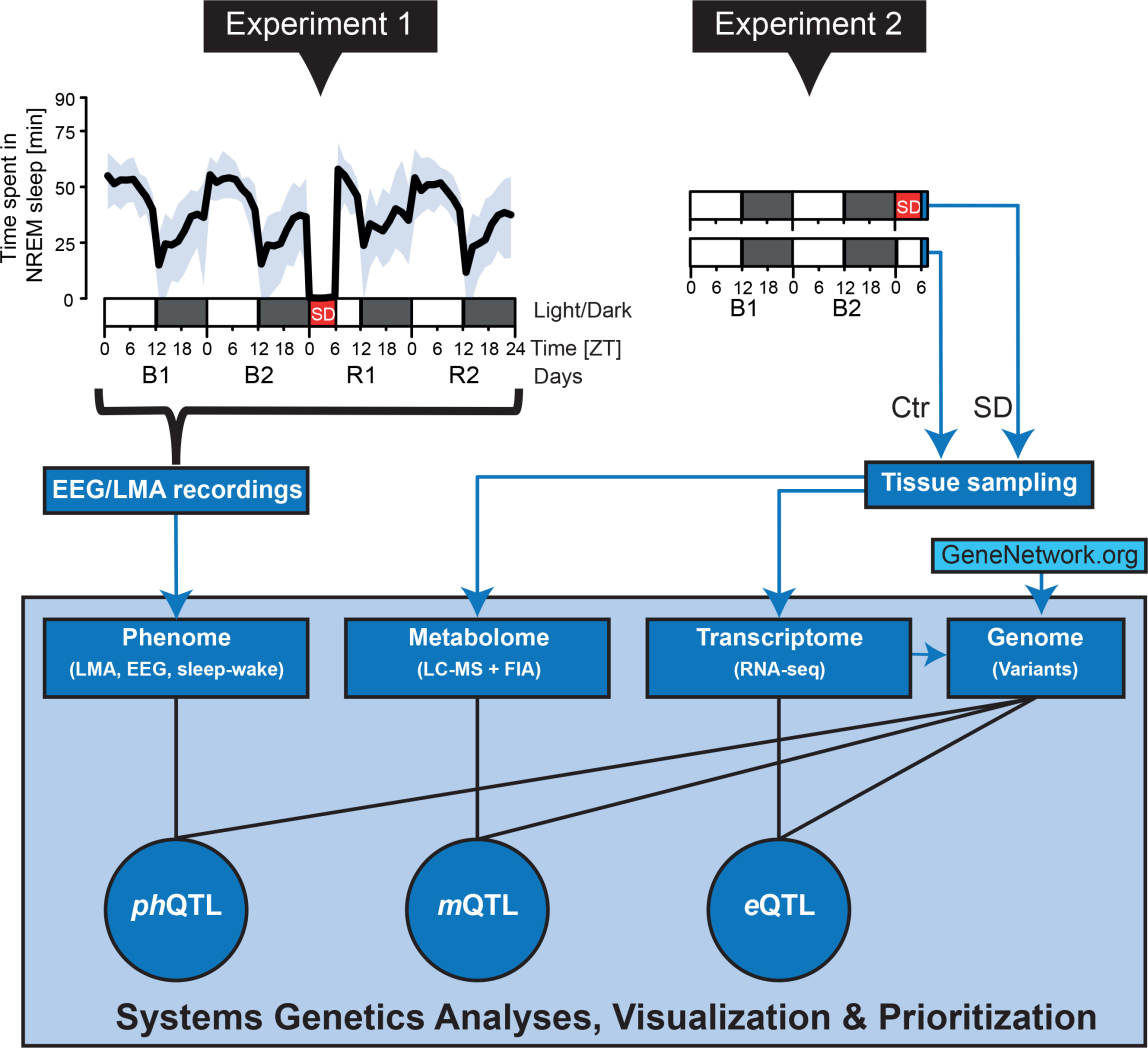
Study design. Thirty-three BXD lines plus the 2 parental strains and their reciprocal F1 progeny were phenotyped. Mice were submitted to either one of 2 experiments. In Experiment 1 (left), EEG/EMG signals and LMA were recorded under standard 12:12 h light–dark conditions (white and black bars under top-left panel) for 2 baseline days (B1, B2), a 6 h SD (red bar) from ZT0–6 (ZT0 = light onset), followed by 2 recovery days (R1, R2). The deep sleep-wake phenome consists of 341 sleep-wake state-, LMA-, and EEG-related phenotypes quantified in each mouse, among which time spent in NREM sleep (gray area spans mean maximum and minimum NREM sleep time among BXD lines, respectively, for consecutive 90 min intervals). Mice in Experiment 2 (right) were used to collect cortex, liver, and blood samples at ZT6. Half of the mice were challenged with an SD as in Experiment 1, the other half were left undisturbed and served as controls (labeled Ctr). Cortex and liver samples were used to quantify gene expression by RNA-seq, blood samples for a targeted analysis of 124 metabolites by LC/MS, or with FIA/MS. For *ph*QTLs, *m*QTLs, and *e*QTLs, a high-density genotype dataset (Genome; approximately 11,000 SNPs) was created, merging identified RNA-seq variants with a publicly available database (www.genenetwork.org). The entirety of the multilevel dataset was integrated in a systems genetics analysis to chart molecular pathways underlying the many facets of sleep and the EEG, using newly developed computational tools to interactively visualize the results and pathways, and to prioritize candidate genes. EEG/EMG, electroencephalography/electromyogram; *e*QTL, expression quantitative trait locus; FIA/MS, flow injection analysis/mass spectrometry; LC/MS, liquid chromatography/mass spectrometry; LMA, locomotor activity; *m*QTL, metabolic quantitative trait locus; NREM, non-REM; *ph*QTL, phenotypic quantitative trait locus; RNA-seq, RNA sequencing; SD, sleep deprivation; ZT, zeitgeber time.

We subjected mice from 33 BXD/*RwwJ* lines (see https://bxd.vital-it.ch; Downloads, General_Information.xlsx for a listing), the 2 parental strains (B6 and D2), and F1 individuals from reciprocal crosses between the parental lines to a deep behavioral and molecular phenotyping across 4 levels of organization. In 1 set of mice, we recorded sleep-wake behavior, brain activity (by EEG), and LMA for 4 d (Fig 1, Experiment 1). On day 3, mice underwent an SD challenge during the first 6 h of the light period, when mice normally sleep most of the time. During SD, an average of 8.6 ± 0.7 successful attempts at sleep were observed lasting 14.2 ± 0.6 s on average, resulting in a total of 1.8 ± 0.1 min (range: 0.0–9.8 min, *n* = 198 over the 33 BXD lines) of sleep or 0.5% of the 6 h intervention. Both the number of sleep episodes and total time spent asleep varied according to BXD line (1-way ANOVA, *p* < 0.0001 for both variables), while response time of the experimenter (i.e., episode duration) did not (*p* = 0.66). Aided by a specifically developed, supervised machine learning–based algorithm (see Materials and methods and S1 Fig), we could extract a comprehensive set of EEG/behavioral phenotypes (see https://bxd.vital-it.ch; Downloads, General_Information.xlsx), which were separated into 3 main biological categories related to (i) LMA, (ii) EEG signal features, and (iii) the prevalence and time structure of sleep-wake state, collectively referred to as “LMA,” “EEG,” and “State,” respectively. The 3 phenotypic categories were divided further into subcategories (see Materials and methods) and by experimental condition (baseline, SD, and recovery). Because some of the 341 phenotypes we quantified were tightly linked (e.g., the time spent in non-REM [NREM] sleep and wakefulness), we estimated the total number of distinct phenotypic clusters or modules to be 120 or 148 when considering phenotypes of different subclasses (e.g., “EEG,” “State,” or “LMA”) within a given module as separate (S2 Fig, Materials and methods, and https://bxd.vital-it.ch; Downloads, General_Information.xlsx). Most phenotypes were unique or were grouped in modules of 2 phenotypes only (67%; median: 2 phenotypes/module, range: 1–13). Several of these modules (49/120) were associated into 3 larger “superclusters” (Supercluster I–III; S2 Fig), containing 18, 20, and 11 modules, respectively. Supercluster I grouped almost exclusively “State”-related phenotypes (80/83), while Supercluster II was composed mostly of “EEG”-related phenotypes (65/73). Supercluster III was composed of 10 “LMA”-related and 30 “State”-related phenotypes. However, in our analyses, we still used all available phenotypes to detect potential regulatory differences among even closely related phenotypes and to avoid analysis bias arising from selecting a “representative” phenotype.

A second set of mice, representing the same lines, was processed in parallel for collection of brain, liver, and plasma (Fig 1, Experiment 2) to measure gene expression in cortex and liver and metabolites in plasma. These transcriptomic and metabolomic data are collectively referred to as (intermediate) molecular phenotypes. We quantified 124 metabolites (see https://bxd.vital-it.ch; Downloads, General_Information.xlsx) using targeted metabolomics covering 5 important metabolite classes (i.e., amino acids, biogenic amines, acylcarnitines, sphingolipids, and glycerophospholipids). Cortex and liver transcript levels were measured using RNA sequencing (RNA-seq), and we detected about 14,900 expressed genes in the cortex and about 14,100 genes in the liver after filtering and normalization.

We used the RNA-seq alignments also to genotype the lines to verify that no mix-up occurred during the breeding and data collection phase, and to increase mapping resolution. We compared the around 500,000 detected genotypes with the publicly available 3,500-genotype set for the same BXD lines from GeneNetwork (2005 release; see Materials and methods). We observed only an approximately 1% discrepancy and merged both genotype sets, resulting in a set of about 11,000 tag variations, which increased the number of haplotype blocks from 551 (GeneNetwork) to 1,071 (RNA-seq + GeneNetwork). All analyses we report here were based on our merged map (see https://bxd.vital-it.ch; Downloads, Genotypes.GeneNetwork2005AndRNAseq.geno). Of note, by the completion of this publication, an updated set of BXD genotypes was released with an estimated haplotype block number of 816 for the specific lines we used (GeneNetwork, 2017 release http://genenetwork.org). Of the 61 significant phenotypic quantitative trait loci (*ph*QTLs) we detected (see below), 54 were also detected using either GeneNetwork genotypes (the 2005 or 2017 release), while the remaining 7 significant *ph*QTLs were unique to our merged genotype map.

### Heritability and QTLs

**Fig 2:**
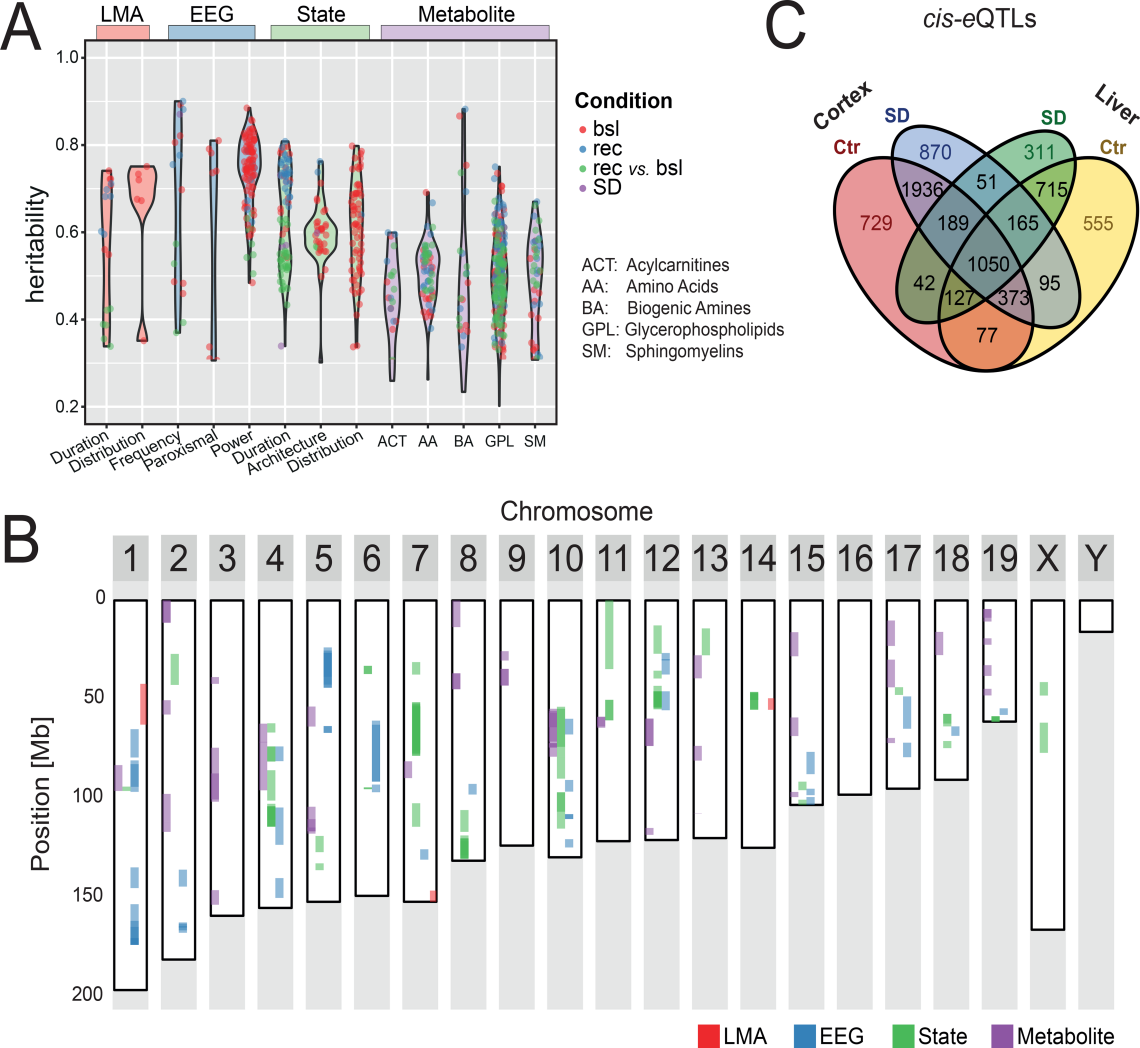
Genetic diversity in the BXD panel greatly impacts behavioral, metabolic, and molecular traits. The phenome was divided into 3 phenotypic categories: (i) LMA, (ii) EEG features (labeled EEG), and (iii) sleep-wake state characteristics (labeled State), which were subdivided further (see Materials and methods). The 5 classes of metabolites and the gene expression represent intermediate molecular phenotypic categories. (A) Heritability for EEG/behavioral and metabolite phenotypes. Dots represent single phenotypes within each category and subcategory indicated along the x-axis. Red dots represent phenotypes recorded in baseline (labeled bsl; B1 and B2), blue in recovery (labeled rec; R1 and R2), purple during SD, and green dots refer to the recovery-to-baseline contrasts. Values represent narrow-sense heritability. (B) Overview of significant and highly suggestive (FDR < 0.1) QTLs obtained for all 341 EEG/behavioral phenotypes (*ph*QTLs: LMA in red, EEG in blue, and sleep-wake state in green) and 124 blood metabolite levels in baseline and recovery (*m*QTLs; purple). Note that overlap of neighboring QTLs renders color shading darker. (C) Venn diagram of genes under significant *cis-e*QTL effect in liver and cortex for the two experimental conditions (SD and controls [labeled Ctr]). EEG, electroencephalography; *e*QTL, expression quantitative trait locus; FDR, false discovery rate; LMA, locomotor activity; *m*QTL, metabolic quantitative trait locus; *ph*QTL, phenotypic quantitative trait locus; QTL, quantitative trait locus; SD, sleep deprivation.

To obtain a first sense of the contribution of genetic factors to the phenotypic variability contained within our BXD set, we examined the heritability of the EEG/behavioral and metabolic phenotypes. The estimated narrow sense heritability [26] among the EEG/behavioral phenotypes was high overall (median h^2^ = 0.68, Fig 2A), consistent with what has been reported in previous human and mouse studies [27]. We also confirm that various aspects of the EEG signal are among the most heritable traits with, in our dataset, theta-peak frequency (TPF) in REM sleep ranking highest (h^2^ = 0.89). The heritability for differential EEG/behavioral phenotypes (i.e., recovery versus baseline; green symbols in Fig 2A) were consistently lower by around 0.2 points compared with the heritabilities obtained for recovery or the baseline values per se. By contrasting individual recovery values to the baseline strain averages, instead of to each animal’s individual baseline value (thereby keeping within strain variance similar to that of the absolute recovery values), we found that this effect did not simply reflect increased variability due to combining recovery and baseline values and thus suggests a smaller genetic contribution to the response to sleep loss. The overall heritability of plasma metabolite levels was somewhat lower than for EEG/behavioral phenotypes (median h^2^ = 0.50), with alpha-aminoadipic acid (α-AAA) displaying the highest heritability (h^2^ = 0.88; Fig 2A).

Average-to-high heritabilities are a requirement to attribute phenotypic variation to gene loci, but even then, there is no guarantee to find genome-wide significant QTL(s); e.g., for the TPF in REM sleep phenotype mentioned above, only 4 suggestive *ph*QTLs of small effect size were identified (see https://bxd.vital-it.ch; Downloads, QTL_Mapping.xlsx) that together could nevertheless account for 58% of the variance (estimated using an additive model, see Materials and methods), suggesting that perhaps higher-order loci interactions (e.g., epistasis), which cannot be captured using the single-marker linkage analysis we used here, underlie differences in this EEG trait. Genome scans revealed a total of 61 “significant” (false discovery rate [FDR] ≤ 0.05), 65 “highly suggestive” (0.05 < FDR ≤ 0.10), and 923 “suggestive” (0.10 < FDR ≤ 0.63) [28,29] *ph*QTLs and 21 significant, 40 highly suggestive, and 528 suggestive metabolic quantitative trait loci (*m*QTLs; Fig 2B).

Several phenotypes from distinct phenotypic categories were associated with overlapping genomic regions. For example, differences in baseline wake consolidation, gain in REM sleep time after SD, EEG delta power (1.0–4.0 Hz) in REM sleep, baseline levels of serotonin and phosphatidylcholine acyl-alkyl (PC-aa)-C34:4, and levels of PC-aa-C34:4 and PC-aa-C36:6 after SD all mapped to one 30 Mb region on chromosome 10 (50–80 Mb), each with a significant or highly suggestive QTL (Fig 2B). These overlapping QTLs may point to pleiotropic effects of 1 underlying gene or close but distinct underlying QTLs.

We also performed QTL analysis for gene expression, but because many more linkage tests were required for transcriptome mapping, we used a more suitable method than for *ph*- and *m*QTL mapping. The format for reporting expression quantitative trait loci (*e*QTLs) will therefore differ from that used for *ph-* and *m*QTLs (see Materials and methods). The expression of individual genes was mapped separately for *cis-e*QTLs with genetic markers within a 2 Mb window and *trans-e*QTLs with markers positioned throughout the genome (see Materials and methods). The transcriptome of BXD mice showed strong linkage with genotypic variation. For example, in the cortex, the expression of 5,704 genes (i.e., 38% of all expressed genes) was significantly driven by a *cis*-variation (Fig 2C and https://bxd.vital-it.ch; Downloads, cis_eQTL.xlsx). Moreover, 2,465 (34%) of all genes under *cis*-*e*QTL effect in both tissues passed the 0.05 FDR cutoff in a single condition and tissue. Factors contributing to this tissue/condition specificity are the absence of gene expression in one of the 2 tissues or a different gene regulatory environment on which SD had pervasive effects (see Pervasive effects of SD at all levels). This important tissue/condition specificity also applied to *trans*-*e*QTLs with 5,537 (53% of 10,450) being under *trans*-*e*QTL effect only in one specific tissue or condition. Although the observation that a large portion of *e*QTLs reached significance in 1 tissue and condition only does suggest widespread gene × environment interactions regulating gene expression, reaching the 0.05 FDR threshold or not does not prove this. We therefore compared linkage strength of significant *cis-e*QTLs that were specific for 1 tissue and condition with that in the 3 other RNA-seq sets. Among the 870 genes with a significant *cis-e*QTL effect in sleep-deprived cortex only (Fig 2C), 175 (20%) showed a significant difference in linkage signal (FDR < 0.05). This proportion was similar in the control cortex and liver (19% and 21%, respectively) and somewhat higher in sleep-deprived livers (32%).

### Systems genetics visualization

**Fig 3:**
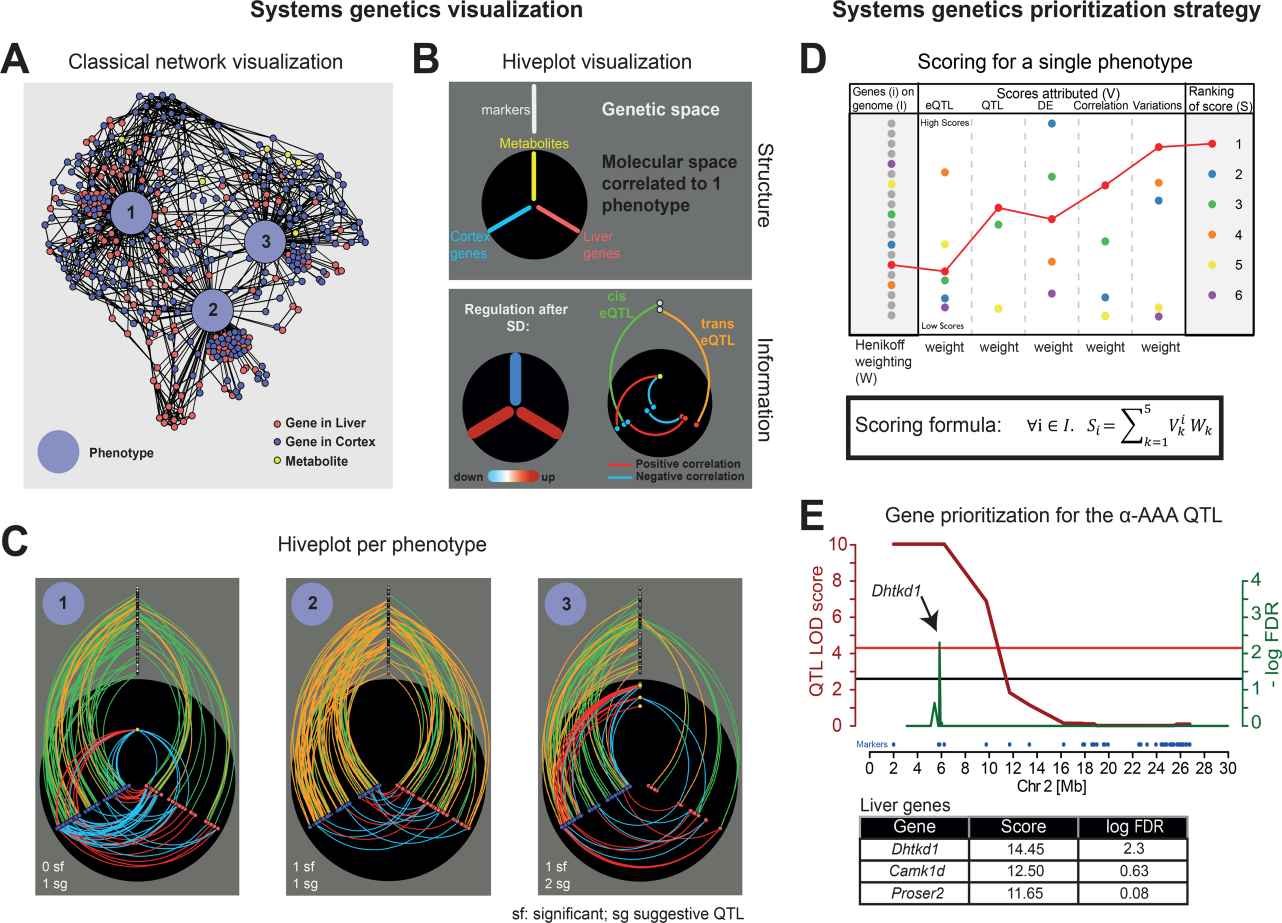
How to visualize multidimensional networks and prioritize candidate genes? (A) Classical network visualization methods strongly depend on the layout algorithm used for positioning nodes, making structure interpretation and reproducibility difficult. (B) Hiveplot network visualization and structure strategy. See text for details. (C) The classical network visualization for the 3 phenotypes (blue nodes 1–3) in panel A can be represented with our method with 1 hiveplot per phenotype. Phenotype 1 showed more cortex–liver correlations than the 2 other phenotypes through 1 metabolite, connecting up- and down-regulated genes in cortex after SD and down-regulated genes in liver. Phenotype 2 shows genomic regions with strong allelic effect over multiple genes in liver and cortex through a high number of *trans-e*QTLs. Phenotype 3 was mostly connected to cortically expressed genes correlating strongly with up-regulated metabolites; most *cis/trans-e*QTLs affected only cortical genes. The number of significant (labeled sf) and suggestive (labeled sg) *ph*QTLs detected for each phenotype are indicated on bottom left. The 3 phenotypes were related to active wake behaviors during recovery (Phenotype 1 and 2: LMA per hour awake and time in TDW, respectively, both during ZT12–24; Phenotype 3: Gain in time spent awake during ZT24–6). (D) Gene prioritization strategy to identify candidate genes associated with phenotype/metabolite variation, illustrated for 6 genes. Five types of analyses were integrated into a single score for each gene to reflect its strength as candidate gene, namely from left to right (i) and (ii) QTL mapping for gene expression (*e*QTLs) and *ph*- or *m*QTLs, respectively, (iii) DE after SD, (iv) gene expression/phenotype correlations, and (v) analysis of protein-damaging genetic variations relating genes to an allelic effect. See text for further details. (E) To illustrate and validate our scoring, strategy, genes in liver were prioritized for levels of α-AAA after SD. *Dhtkd1* was identified as top-ranked candidate gene. Results from QTL mapping (red line) and prioritization analysis (green line); red and black horizontal lines indicate significant thresholds for the QTL and prioritization, respectively. α-AAA, alpha-aminoadipic acid; DE, differential expression; *e*QTL, expression quantitative trait locus; FDR, false discovery rate; LMA, locomotor activity; LOD, logarithm of odds ratio; *m*QTL, metabolic quantitative trait locus; QTL, quantitative trait locus; *ph*QTL, phenotypic quantitative trait locus; SD, sleep deprivation; TDW, theta-dominated waking; ZT, zeitgeber time.

The complexity of multilevel networks can only be appreciated through visual aids. Because the widely used “hairball” representation, in which biological factors are represented as “nodes” and their interconnections as “edges,” is hardly interpretable due to its nondeterministic structure (Fig 3A), we opted for a structured representation more suitable for the visualization of complex systems, namely, “hiveplots” [25]. The hiveplots were laid out as follows: each plot represents 1 EEG/behavioral phenotype and its associated molecular network—i.e., only the genes and metabolites strongly correlated with a given phenotype are displayed (Fig 3B; see Materials and methods for details). Each hiveplot is composed of 3 radial axes containing the molecular data with nodes assigned to the 2 bottom axes for genes expressed in the cortex (Fig 3B left, in blue) and liver (Fig 3B right, in red), while nodes on the vertical axis (Fig 3B top, in yellow) represent metabolites. On top, we added a separate “genetic” axis (Fig 3B top, white) containing the genotypes. The node position on the 3 (molecular) radial axes was determined by the response to SD—i.e., molecules positioned closer to the center were down-regulated more strongly, while more up-regulated genes/metabolites can be found closer to the axes’ perimeter. Edges connecting nodes represent positive/negative correlations (red/blue, respectively) between measurements of expression/metabolite levels. Genetic markers linked to genes by *e*QTLs connect the genetic and molecular space.

The hiveplot representation allows investigation of the molecular network associated with an EEG/behavioral phenotype in a structured manner and comparison of phenotypes using all intermediate phenotypic layers available in the dataset. The difference in presence or absence of nodes/edges between 2 phenotypes indicates which association was gained or lost. Furthermore, the importance of the SD effect on these nodes can be visually estimated by their position along the axis (Fig 3C). Although the interphenotype connectivity present in the hairball representation is lost in the printed format of these hiveplots, this aspect can be easily accessed through our web interface (https://bxd.vital-it.ch) by highlighting common edges. The web interface also allows for an in-depth exploration of the data by displaying node details, such as gene and metabolite name, and variation identifiers. It also lets the user modify the parameter settings, such as the correlation strength used to include correlated genes and metabolites, with which the hiveplots are generated (see S3 Fig and the tutorial on https://bxd.vital-it.ch; Help).

### Systems genetics prioritization

We developed an unbiased, data-driven approach to select candidate genes associated with our EEG/behavioral and metabolic phenotypes. We focused on genes located in the associated genomic regions found by QTL analyses (see Fig 2B). To investigate these often quite large regions (mean = 9.8 Mb, range = 0.7–34.7 Mb for significant and highly suggestive *ph*QTLs), we implemented a scoring strategy inspired by the “similarity profiling prioritization strategy” [30], which combines multiple sources to prioritize a gene. For each gene, we computed an integrated score composed of (i) the genomic position of the gene with respect to the *ph-*/*m*QTL peak, (ii) a detected *cis*-*e*QTL driving the expression of the gene, (iii) a protein-damaging annotation of a variant, (iv) differential expression (DE) after SD, and (v) correlation between expression and phenotype of interest (Fig 3D, S4 Fig, see Materials and methods for details). Our prioritization strategy thus aimed at identifying genes that are sensitive to sleep loss, correlated with the phenotype being evaluated, associated to a *cis*-*e*QTL, and/or carrying a protein-damaging variant that could contribute to trait variance. A Henikoff weighting algorithm was applied to correct for intrinsic correlations among the 5 analysis scores. One informative example of such intrinsic correlation is a *cis-e*QTL located within a *ph*QTL region, in which case the phenotype–gene expression correlation will be influenced by linkage. The algorithm decreases the *cis-e*QTL score accordingly, and *cis-e*QTLs therefore usually contributed with a low score to the prioritization (see S1 Table for examples). The integrated score for each gene was computed with the given formula (Fig 3D), and an FDR was computed by performing 10,000 permutations (S4 Fig and Materials and methods). For each QTL, we kept the gene with the highest significant integrated score. This scoring strategy was applied to cortex and liver data separately.

To illustrate our prioritization algorithm, we applied it to the metabolite with the highest heritability, α-AAA (see above), and for which we obtained a highly significant *m*QTL on chromosome 2 (logarithm of odds ratio [LOD] = 9.25, 1–11 Mb). We readily identified *Dhtkd1* as the top-ranked significant candidate gene in liver within the chromosome 2 *m*QTL (Fig 3E) because of (i) the strong correlation of *Dhtkd1* expression with α-AAA levels, (ii) *Dhtkd1* is under a *cis*-*e*QTL effect (*rs222492362*, chr2: 5.8 Mb, *q* = 1.5e−17), (iii) the marker of the *cis*-*e*QTL is located within the peak of the *m*QTL, and (iv) both α-AAA and *Dhtkd1* levels are affected similarly by SD. The 5 scores and weights of this example and those obtained in Examples 1–4 (see below) are detailed in S1 Table.

This result can be taken as a first validation of our scoring strategy because *Dhtkd1* encodes an enzyme subunit involved in lysine degradation known to control α-AAA levels in BXD lines [31]. Although with this particular example, the prioritization tool did successfully select the causative gene underlying the α-AAA *m*QTL, it is important to note that, as opposed to other tools that have been developed (e.g., [32,33]), our algorithm cannot infer causality and is designed to help select likely candidate genes within *m*- and *ph*QTLs.

### Pervasive effects of SD at all levels

**Fig 4:**
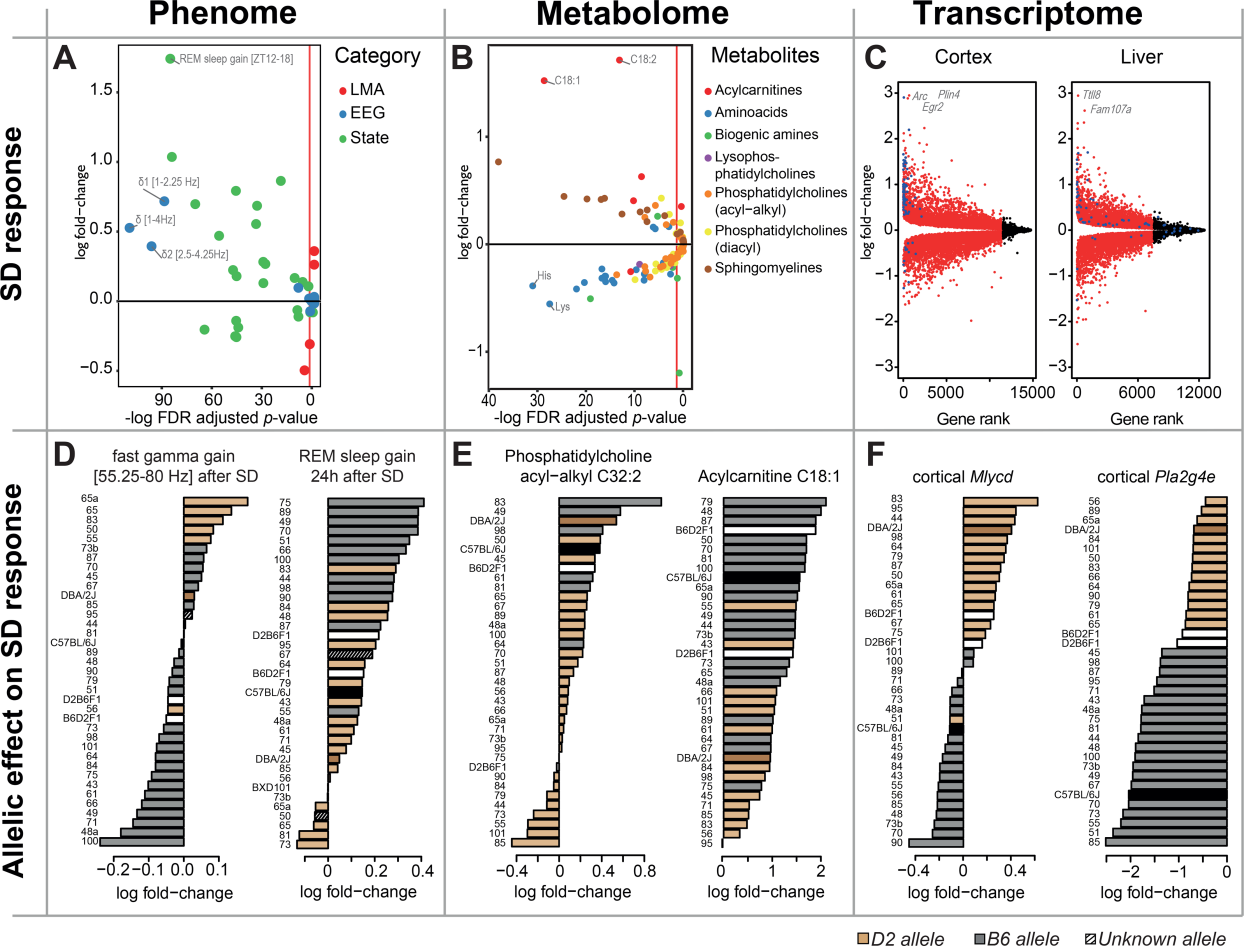
Profound effects of SD on transcriptome, metabolome, and phenome. EEG/behavioral phenotypes, metabolites, and transcripts are organized into 3 “columns” (from left to right). Top 3 panels show the SD response (recovery/baseline fold change). Bottom 3 panels depict examples of allelic effects on the SD responses, with color-coding indicating the presence of a C57BL/6J or DBA/2J haplotype under the mapped QTL peaks (B6: gray for BXD and black for parental; D2: light brown for BXD and dark brown for parental). White bars mark the F1s and hatched bars strain in which haplotype could not be unambiguously determined. (A) Phenotypic changes after SD. The top significantly changed phenotype was the increase in NREM sleep EEG delta power (1–4 Hz) after SD (far-left blue data point). The most up-regulated phenotype was time spent in REM sleep during the first 6 h of darkness (ZT12–18) after SD (highest green data point). (B) Metabolite changes after SD. Most amino acids (blue) were down-regulated and most sphingolipids (brown) up-regulated after SD. The acylcarnitines C18:1 and C18:2 (highest red dots) increased the most. Vertical red line: significant threshold (FDR-adjusted *p*-value = 0.05). (C) DE analysis (SD/Ctr) for cortex (left) and liver (right). Genes were sorted according to their ranked *p*-value along the x-axis. Significantly affected transcripts in red (FDR-adjusted *p*-value < 0.05), nonsignificant results in black. Blue dots indicate 78 genes considered core molecular components of the sleep homeostatic response in the cortex [34]. Note that no low fold change threshold was applied. (D-F) Examples of genetically driven EEG/behavioral, metabolic, and transcriptional responses to SD, respectively. See text for details. *Arc*, *activity-regulated cytoskeletal-associated protein*; Ctr, control; DE, differential gene expression; EEG, electroencephalography; *Egr2*, *early growth response 2*; *Fam107a*, *family with sequence similarity 107*, *A*; FDR, false discovery rate; LMA, locomotor activity; *Mlycd*, *malonyl-CoA decarboxylase*; NREM, non-REM; *Plin4*, *Perilipin 4; Pla2g4e*, *phospholipase A2*, *group IVE*; QTL, quantitative trait locus; SD, sleep deprivation; *Ttll8*, *tubulin tyrosine ligase-like family 8*; ZT, zeitgeber time.

The EEG/behavioral and molecular phenotypes were assessed both under undisturbed baseline conditions and after 6 h SD. SD profoundly and significantly impacted a majority of measurements at all levels. We observed the well-known increase in EEG delta power (1.0–4.0 Hz) during NREM sleep as well as the increase in the time spent asleep (Fig 4A), both reflective of an accumulated homeostatic sleep pressure during SD. The gain in time spent in NREM sleep was strongest during the initial 12 h following the SD, with an average gain of +23 min (compared with values reached during corresponding baseline hours) during the first 6 h after the SD (zeitgeber time [ZT]6–12) and +32 min during the first 6 h of the following dark period (ZT12–18). The most strongly affected sleep phenotype concerned time spent in REM sleep, which displayed a 3.3-fold gain during the first 6 h of darkness (ZT12–18) after SD (Fig 4A). SD thereby doubled the proportion of REM sleep to NREM sleep in this interval. Locomotor activity and waking phenotypes were generally decreased during the light period immediately following the SD (ZT6–12).

In addition, the plasma metabolome was profoundly altered by SD. Of the 124 measured metabolites, 75 (60%) were significantly up- or down-regulated. The levels of all amino acids were significantly altered after SD, the majority being down-regulated, with the exception of glutamine, glutamate, and tryptophan, which were up-regulated (Fig 4B). A recent publication reported similar effects on amino acid levels in brain dialysates of sleep-deprived rats [35], suggesting that plasma can report on central changes in amino acid levels. By contrast, tryptophan was the only amino acid that was found significantly changed during SD in humans using the same methodology [36]. The 2 acylcarnitines present in our dataset (C18:1 and C18:2) were both strongly up-regulated with a greater than 2-fold change. Similar results were found in humans, with acylcarnitines levels increased in blood and carnitines increased in urine after sleep loss [36,37].

The transcriptome was especially sensitive to SD, with 78% of all expressed genes being differentially expressed in cortex and 60% in liver. In cortex, the most strongly differentially expressed genes were *activity-regulated cytoskeletal-associated protein* (*Arc*), *early growth response 2* (*Egr2*), and *perilipin 4* (*Plin4*), with an almost 8-fold increase in expression after SD (see S2 Table). *Arc* is an immediate early gene crucial for long-term synaptic plasticity and memory formation [38]. *Arc* is among the most consistently up-regulated transcripts after SD [39] and features in a short list of 78 genes, the expression of which we found reliably and significantly changed by extended wakefulness under a number of experimental conditions [34]. Forty-nine other genes in this short list featured among the top 5% most affected transcripts of the current experiment (S2 Table and blue symbols in Fig 4C left; enrichment *p* = 5.6e−43, Fisher test). The remaining 29 of this short list were all significantly affected by SD also in the current study, 15 of which were found in the 5%–10% tile, and all ranked in the top 26% of most differentially expressed genes. Similarly, *Egr2* is 1 of 3 *Egr* genes that are rapidly induced by SD in several species [39]. *Egr1* and *Egr3* appear on our short list of 78, and all 3 *Egr*s are among the top-100 differentially expressed cortical genes in the current study (S2 Table). The *Egr* family are immediate early genes encoding transcription factors important in neuronal plasticity [40]. *Plin4*, which encodes a lipid droplet–associated protein involved in lipid storage [41], has not been reported previously as part of the SD response. *Tubulin tyrosine ligase-like family 8* (*Ttll8*), encoding a ligase that glycylates microtubules [42], and *family with sequence similarity 107*, *A* (*Fam107a*), a stress- and glucocorticoid-regulated gene [43,44], were the top differentially expressed genes in liver (S3 Table). Although the short list of 78 was based on forebrain samples, 17 genes were also present in the top 5% differentially expressed genes in the liver (blue symbols in Fig 4C right). Moreover, 13 genes were common to the top 5% list in cortex, liver, and the 78 genes of the short list (*Hspa1a*/*b*, *Cirbp*, *Fos*, *P4ha1*, *Chordc1*, *Dusp1*, *Slc5a3*, *Hsph1*, *Creld2*, *Tra2a*, *Zbtb40*, and *Pfkfb3*). These genes might be interesting candidates for tissue-independent biomarkers of sleep pressure.

### Genetics of the effects of SD

In the context of our project, a key question is whether genetic background modifies these pervasive effects of SD. We found evidence for this at all 3 levels of organization and detected genomic loci predicting differences not only in the magnitude of the response to SD but also in the direction of the response (illustrated in Fig 4D–4F). In the analyses, we included both the levels reached after the SD and these levels contrasted with their baseline levels. These contrasts will be referred to as “change,” “increase,” “gain,” “decrease,” or “DE”.

For 7 EEG/behavioral “gain” phenotypes we discovered a significant QTL (https://bxd.vital-it.ch; Downloads, QTL_Mapping.xlsx). Illustrated in Fig 4D is the gain in time spent in REM sleep, which mapped significantly to chromosome 18 (LOD = 3.9; 57–62 Mb) with B6-allele carriers gaining more REM sleep than D2-carriers (genotype × SD interaction: *p* = 2.0e−5). Three more “gain” phenotypes will be discussed in detail below (see Example 1, 3, and 4 in the Systems genetics of the effects of SD section). Also illustrated in Fig 4D is an EEG/behavioral gain phenotype with a pronounced genotype effect on the direction of change. The SD-induced changes in EEG activity in the fast gamma band (55–80 Hz) in NREM sleep mapped suggestively to chromosome 6 (LOD = 2.83; 77–89 Mb), with a majority of B6-allele carriers at the QTL peak position having a significant decrease in fast gamma, while several D2-allele carriers showed a significant increase (genotype × SD interaction: *p* = 1.0e−5).

Examples of 2 genetically driven metabolic responses to SD are illustrated in Fig 4E. The change in PC-ae-C32:2 after SD mapped significantly to chromosome 5 (LOD = 3.6; 58–69 Mb; genotype × SD interaction: *p* = 2.0e−3). The change in acylcarnitine C18:1, the strongest among all metabolites assayed (Fig 4B), mapped suggestively to chromosome 18 (LOD = 3.6; 73–75 Mb; genotype × SD interaction: *p* = 2.0e−3). For an additional 79 metabolites, a significant genotype × SD interaction was obtained that mapped at the suggestive level (see https://bxd.vital-it.ch; Downloads, Genotype_SD_Interaction.xlsx). Finally, significant *cis-e*QTLs were detected for the DE (i.e., recovery versus control) of 195 genes after SD in cortex and 62 in liver (see https://bxd.vital-it.ch; Downloads, Genotype_SD_Interaction.xlsx and cis_eQTL.xlsx). The strongest *cis-*allele in cortex was found for the DE of *phospholipase A2*, *group IVE* (*Pla2g4e*; *rs47077493*, chr2: 118.3 Mb, *q* = 1.2e−9) with a down-regulation that was 2-fold larger in B6- than in D2-allele carriers (genotype × SD interaction: *p* = 1.0e−9; Fig 4F). Also illustrated are the effects of SD on *malonyl-CoA decarboxylase* (*Mlycd*) expression for which a *cis-e*QTL was identified (*rs33610973*, chr8: 120.8 Mb, *q* = 1.9e−5). In BXD lines carrying a B6-allele at the *cis-e*QTL position, a down-regulation of *Mlycd* was observed, while the opposite was true for D2-allele carriers (genotype × SD interaction: *p* = 2.0e−4; Fig 4F). *Pla2g4e* encodes a phospholipase promoting the formation of free fatty acids (FFAs), while *Mlycd* encodes an enzyme promoting mitochondrial fatty acid oxidation. One last example of a significant differential *cis-e*QTL, for *Werner syndrome RecQ like helicase* (*Wrn*), will be discussed in detail below (see Example 1 in the Systems genetics of the effects of SD section). It should be noted that for most of the significant differential *cis-e*QTLs, including *Wrn*, DE and the absolute expression after SD were highly correlated (>0.5; 140/195 in cortex), and both were regulated by shared *cis-e*QTLs (161/195).

### Systems genetics of the effects of SD

In the following 4 sections, we highlight 4 phenotypes quantified during recovery from SD that emerged from our systems genetics analyses because of the presence of strong genetic evidence at all levels of organization. Two concern the levels of EEG delta power reached after SD, 1 concerns the gain in time spent in NREM sleep during recovery, and, as a last example, the changes in TPF during REM sleep in recovery. While for the first 3 phenotypes abundant evidence exists documenting their change with SD and their relevance in optimal daytime functioning and health, the latter phenotype (which has not been reported on previously) illustrates that, depending on genotype, a phenotype can either increase or decrease after sleep loss. Moreover, this example shows that phenotypes considered strictly “central” (i.e., the frequency of hippocampal theta oscillations) are strongly associated with genomic loci affecting gene expression in the periphery and not in the brain. It is important to point out that the genomic loci identified for these 4 recovery phenotypes appear after SD only and not (even at the suggestive level) under baseline conditions. Of equal importance is pointing out that our analyses cannot provide causal proof; instead, the systems genetics approach’s power lies in generating new hypotheses that need experimental confirmation. A first step in that direction was made in Example 4 below.

#### Example 1: Genetic heterogeneity in the gain of slow and fast EEG delta power after SD

The prevalence and amplitude of EEG oscillations in the delta frequency range (1.0–4.0 Hz) during NREM sleep can be quantified as EEG delta power. The sleep-wake-dependent changes in EEG delta power have been widely used as a marker of the sleep homeostatic process and form the basis of leading hypotheses on sleep-wake regulation and function [4,6,45]. The sleep-wake-dependent changes do not, however, affect all delta frequencies to the same extent, and therefore, the presence of slow (δ1) and fast (δ2) delta bands have been recognized in humans, rats, and mice, each with different dynamics and different response to experimental interventions [46–51]. As a neurophysiological correlate of the increased EEG activity in δ1 activity after SD, increased noradrenergic tone in the cortex has been proposed [52], while the acceleration of the clocklike delta oscillations generated by thalamocortical neurons at increasing levels of hyperpolarization that accompany deep NREM sleep could contribute to increases in δ2 activity [53,54]. Although the various studies used different frequencies ranges to delineate the δ1 and δ2 delta bands, we here used the 1.0–2.25 and 2.5–4.25 Hz bands, respectively, according to our previous publications [46,47].

**Fig 5:**
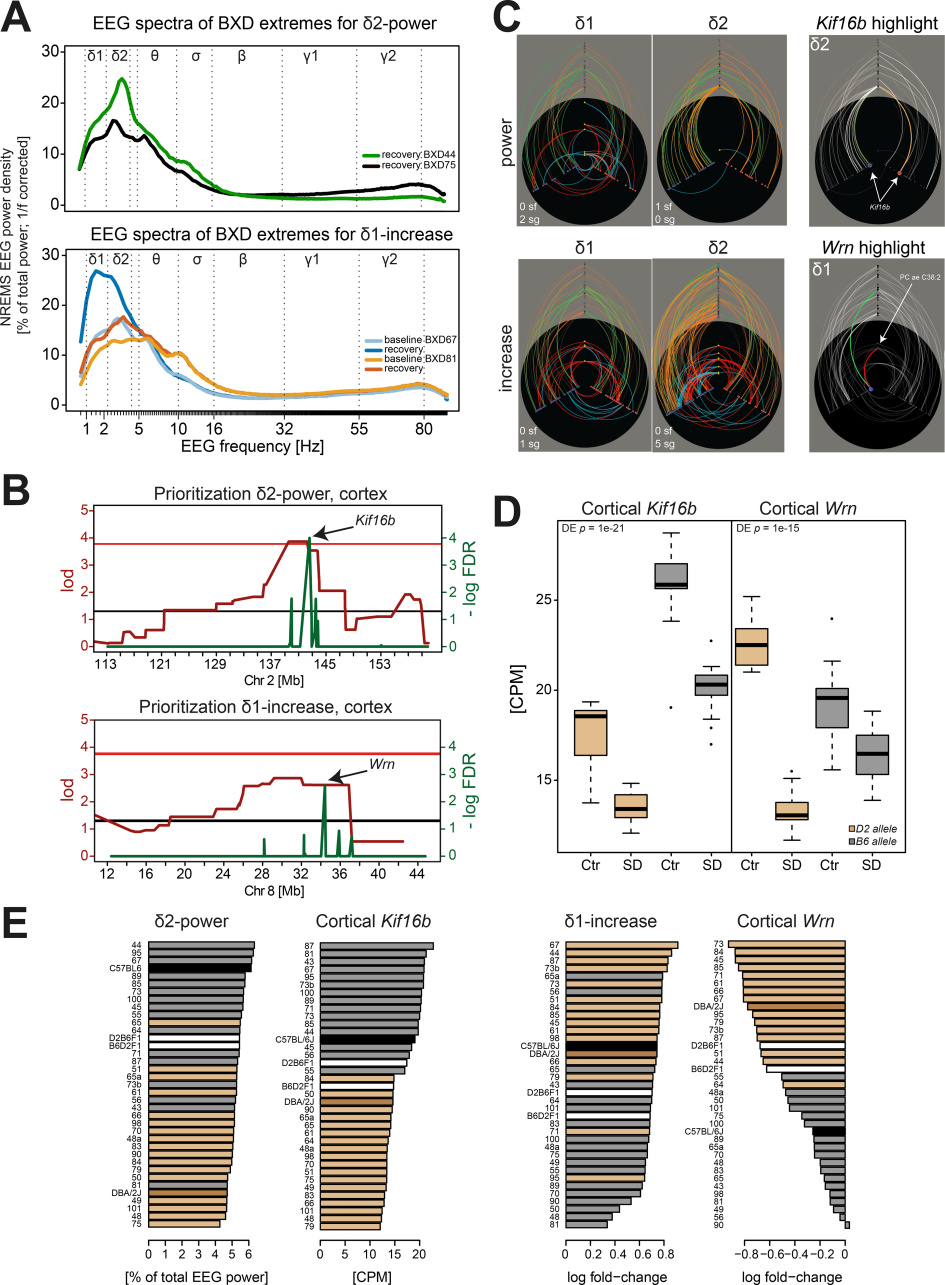
EEG delta power in NREM sleep after SD is associated with *Kif16b* and *Wrn*. (A) NREM sleep EEG spectra in the first 3 h after SD (ZT6–9) for the 2 BXD lines that displayed the lowest and highest EEG activity in the fast delta frequency band (2.5–4.25 Hz, δ2; top, see panel E) and for the 2 BXD lines that displayed the smallest and largest increase (or gain) in EEG power in the slow delta band (1.0–2.25 Hz, δ1; bottom, see panel E). Spectra were “1/f-corrected” (and therefore not directly comparable to the values in panel E) for better visualization of activity in higher frequency bands (theta [5–9 Hz, θ], sigma [11–16 Hz, σ], beta [18–30 Hz, β], and slow [32–55 Hz, γ1] and fast gamma [55–80 Hz, γ2]). Subsequent analyses were performed without this correction. (B) QTL mapping and prioritization for δ2 power identified a significant association on chromosome 2 and *Kif16b* in cortex as top-ranked gene (top). For the δ1 increase after SD, we obtained a suggestive QTL on chromosome 8 and a significant prioritization score for the DNA-helicase *Wrn*. (C) Hiveplot visualization of network connections for the δ1 and δ2 power after SD (top-left panels) and the SD-induced increase in δ1 and δ2 power over baseline (bottom-left panels). Note the marked differences in the networks and QTLs regulating the expression of these 2 delta bands. Right hiveplots highlight *Kif16b* in the δ2 power–associated network (top), and *Wrn* in the network associated with the δ1 increase (bottom). Only *Kif16b* expression in the cortex was linked to the chromosome 2 *cis*-*e*QTL and was not associated with any metabolite. *Wrn* expression was significantly linked to the chromosome 8 *cis-e*QTL and to the long phosphatidylcholine, PC-ae-C38:5. (D) *Kif16b* is highly significantly down-regulated in cortex (left), while it remains unchanged in liver after SD (*p* = 0.15; not shown). Also, *Wrn* expression was strongly down-regulated by SD in cortex (right) and only marginally so, albeit significantly, in liver (*p* = 0.02; not shown). (E) Strain distribution patterns. BXD lines carrying a *B6-*allele on the chromosome 2–associated region showed higher δ2 power after SD (left) and a significantly higher *Kif16b* expression (*p* = 1.3e−15; second to left) than *D2-*allele carriers. *D2-*allele carriers of the chromosome 8–associated region showed a larger δ1 increase after SD (second to right) as well as a significantly larger decrease in *Wrn* expression after SD (right) than *B6-*allele carriers. For color-coding of genotypes, see Fig 4. CPM, counts per million; Ctr, control; EEG, electroencephalography; *e*QTL, expression quantitative trait locus; FDR, false discovery rate; *Kif16b*, *Kinesin family member 16B*; NREM, non-REM; PC-ae, phosphatidylcholine acyl-alkyl; QTL, quantitative trait locus; SD, sleep deprivation; *Wrn*, *Werner syndrome RecQ like helicase*; ZT, zeitgeber time

In the current dataset, we confirmed that EEG activity in the 2 bands responded differently to SD; e.g., lines that showed the lowest/highest gain in δ1 power (i.e., BXD81 and BXD67, respectively; Fig 5A bottom) only ranked 12th and 23rd (out of 33) for the gain in δ2 power. Moreover, while δ1 power gain clustered with the absolute levels of delta and δ1 reached after SD (S2 Fig), δ2 power gain shared an unrelated phenotypic module with delta power gain. Although we did not find loci with strong linkage for the gain in EEG delta power after SD when analyzed for the entire delta frequency range (see S1 Text), we did identify genetic loci contributing to increases in either δ1 or δ2 power over baseline. While 1 suggestive QTL (LOD = 2.58; chr1: 165–176 Mb) was found using the full 1.0–4.0 Hz band, we detected 1 suggestive QTL on chromosome 8 (LOD = 2.86; 18–37 Mb) for the gain in δ1 power, explaining 33% of the phenotypic variance across the BXD lines, and 5 suggestive QTLs for δ2 power gain, none of which overlapped with the δ1 and “full” delta power gain QTLs. Although each of these 5 QTLs explained only <5% of the total variance in δ2 power gain, combined they explained no less than 75% of the variance (estimated using an additive model; see Materials and methods). These genetic findings extend our previous observations that δ1 and δ2 power gain are regulated through distinct signaling pathways.

Gene prioritization significantly scored the DNA-helicase *Wrn* as a candidate for δ1-power gain, while no significant candidates were found for the full delta gain and for the δ2-power gain, probably due to the low effect size for each of the 5 suggestive QTLs. *Wrn* is located within the suggestive QTL on chromosome 8 (Fig 5B bottom), and its expression was strongly associated with a long phosphatidylcholine (PC-ae-C38:5; Fig 5C bottom). We found that *Wrn* expression in the cortex specifically was driven by a *cis-e*QTL (*rs51740715*, chr8: 35.2 Mb, *q* = 1.9e−7) with D2-allele carriers having higher expression levels than B6 carriers under control conditions. Moreover, this same *cis*-*e*QTL region determined the magnitude of the SD-induced decrease in *Wrn* expression, such that after SD, D2-allele carriers now displayed lower levels than B6-allele carriers (genotype × SD interaction: *p* = 5.2e−10; Fig 5D right). Moreover, a higher gain in δ1 power was associated with a stronger down-regulation of *Wrn* after SD (Fig 5E right).

*Wrn* encodes a DNA-repair protein involved in several aging-related diseases [55] and is regulated by *Sirt1* [56], which, in turn, is involved in redox homeostasis, senescence, and wakefulness [57,58]. Down-regulation of *Wrn* alters redox homeostasis through a metabolic shift, impacts glucose metabolism, and increases oxidative stress [59,60]. *Wrn* helicase mutants also showed up-regulation of long phosphatidylcholines [61] relevant for the significant association between *Wrn* expression and PC-ae-C38:5 we reported above. The down-regulation of *Wrn* after SD and its association with the sleep-wake-dependent changes in EEG delta power raise questions concerning its involvement in the known sleep loss–related increases in oxidative stress [62,63] and the age-related reduction in EEG delta power [64,65].

#### Example 2: The level of fast delta activity in the NREM sleep EEG after SD

Apart from the sleep-wake-driven gain in EEG power in the 3 delta bands discussed in Example 1, the prevalence and magnitude of the delta oscillations per se are under strong genetic control both in human and mouse [66,67]. The capacity to generate widespread synchronized cortical activity in the delta frequency range during NREM sleep and the effects of SD thereon represent 2 unrelated EEG phenotypes governed by different genetic factors [68]. Accordingly, the QTLs associated with the gain in δ1 power and in δ2 power presented in Example 1 did not associate with the levels of δ1 power and δ2 power reached after SD. Moreover, as for the δ1- and δ2-gain phenotypes, the levels in delta power measured after SD differed between the δ1 and δ2 frequency bands and did not cluster together, although both were associated with Supercluster II (S2 Fig). For example, the lowest/highest powers for the δ2 band were found in BXD75 and BXD44, respectively (Fig 5A top), while these 2 lines ranked 2nd and 14th (out of 33) for δ1 power. For δ2 power after SD, a significant QTL was identified on chromosome 2 (LOD = 3.87; 136–144 Mb) that explained 42% of the variance. This QTL was specific for δ2 power and did not associate with δ1 power, for which no QTL was found. We did, however, find a suggestive QTL at the same locus for the power in the full delta band explaining a mere 2% of its variance among the BXD lines. This QTL was specific also for recovery sleep, and no linkage was observed in this region for the absolute δ2 power levels in baseline (https://bxd.vital-it.ch; Downloads, QTL_Mapping.xlsx) nor for the significant recovery/baseline gain in δ2 power discussed above in Example 1.

Our prioritization strategy revealed *kinesin family member 16B* (*Kif16b*) as the top significant candidate gene for δ2 power after SD (Fig 5B top). The high prioritization score was based on the strong *cis*-*e*QTL associated with *Kif16b* expression in both cortex and liver (Fig 5C highlight; novel marker, chr2: 142.4 Mb, *q* = 1.3e−15 in cortex, *q* = 7.13e−5 in liver), the pronounced down-regulation of *Kif16b* expression in cortex after SD (Fig 5D left), and the positive correlation between δ2 power after SD and *Kif16b* expression (Fig 5E left). Lines carrying a B6-allele at the chromosome 2–associated region displayed higher δ2 power after SD and a significantly higher *Kif16b* expression compared to D2-allele carriers (Fig 5E left).

*Kif16b* encodes a kinesin involved in early endosome and receptor transport, including of receptors that play a role in sleep regulation such as fibroblast growth factor (FGF) [69], nerve growth factor (NGF) [70], and ionotropic glutamate (α-amino-3-hydroxy-5-methyl-4-isoxazolepropionic acid [AMPA]) [71] receptors. α-amino-3-hydroxy-5-methyl-4-isoxazolepropionic acid receptor (AMPA-R) levels are sleep-wake driven, associated with changes in EEG delta power, and have been explored as therapeutic targets to counter the deleterious effects of SD on cognition [72–76]. Our results thus corroborate a link between fast delta EEG activity after SD and AMPA-R trafficking and implicate *Kif16b* as a candidate molecular go-between. Of interest, given the large changes in *Arc* expression after SD (reported in the section Pervasive effects of SD at all levels), is that increased *Arc* expression reduces the number of AMPA-Rs through its direct interaction with components of the endocytic pathway, thereby contributing to homeostatic synaptic scaling [38,77]. Whether *Arc*-dependent AMPA-R trafficking through the endocytic pathways involves *Kif16b*’s role in the localization of early endosomes requires further study.

#### Example 3: SD shifts TPF in the REM sleep EEG

**Fig 6:**
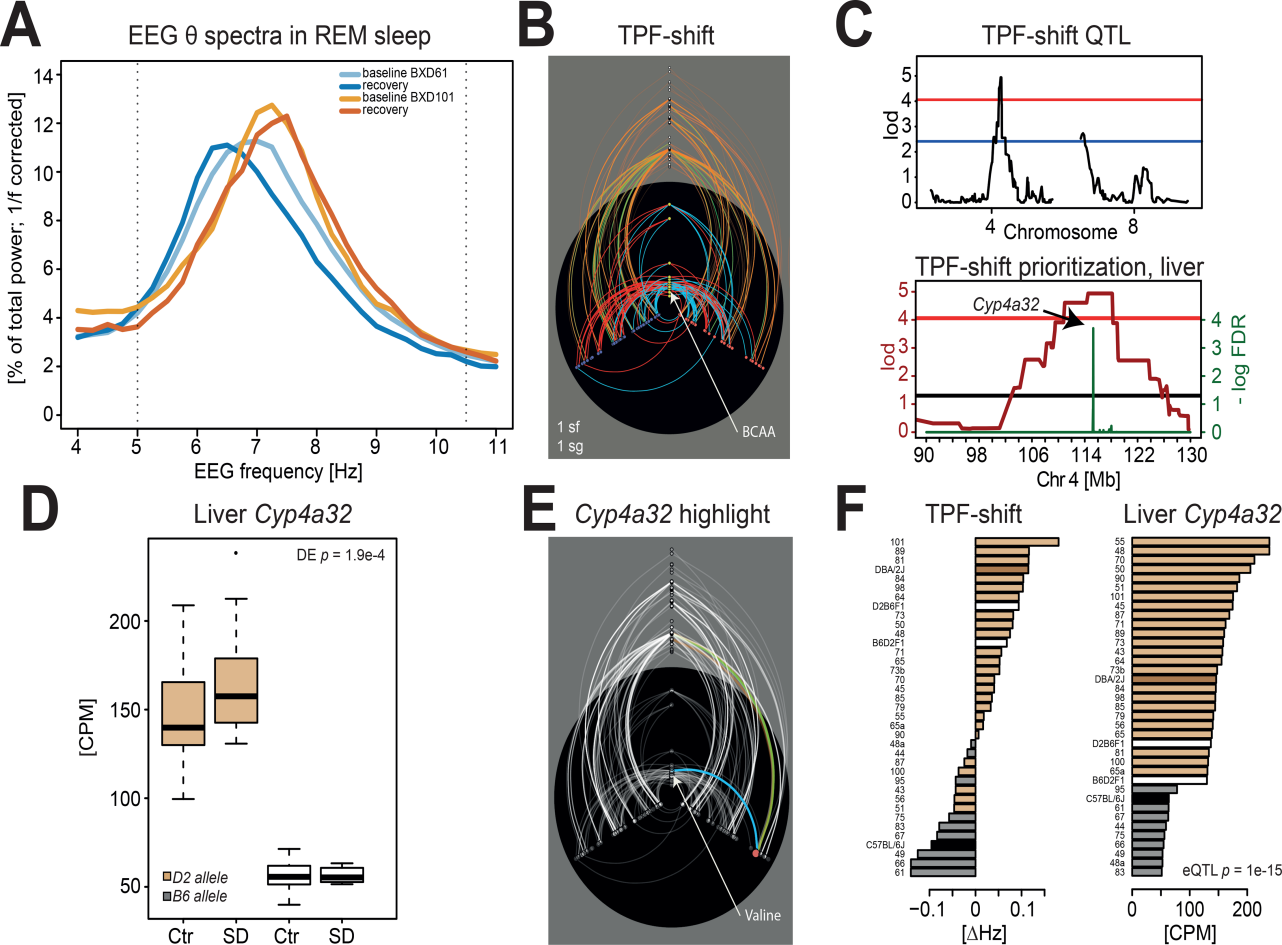
Changes in the frequency of theta oscillation during REM sleep after SD are associated with *Cyp4a32*. (A) Spectral profiles of the REM sleep EEG for 2 strains displaying an opposite shift in the frequency of theta oscillations after SD relative to baseline. This shift was quantified by the decrease and increase in TPF for BXD61 and BXD101, respectively (see panel F). (B) Hiveplot for the SD-induced shift in TPF. (C) One significant QTL for the TPF shift was detected on chromosome 4 and 1 suggestive QTL on chromosome 8. Prioritization yielded *Cyp4a32* as the top-ranked significant gene, based on the significant *cis-e*QTL modifying its expression in liver and a predicted damaging variation (V314E). (D) Effects of SD and genotype on liver *Cyp4a32* expression. Carrying a *B6-*allele at the *Cyp4a32 cis-e*QTL–associated marker greatly decreased its expression. (E) Hiveplot for the SD-induced shift in TPF, highlighting *Cyp4a32*’s links to the amino acid Valine and the chromosome 4 *e*QTL marker. (F) Strain distribution patterns for TPF differences and liver *Cyp4a32* expression after SD. *B6-*allele carriers at the chromosome 4–associated region had lower *Cyp4a32* liver expression and a decrease in TPF after SD, while *D2-*carriers increase TPF and have higher *Cyp4a32* expression. CPM, counts per million; Ctr, control; *Cyp4a32*, *Cytochrome P450*, *family 4*, *subfamily a*, *polypeptide 32*; DE, differential expression; EEG, electroencephalography; *e*QTL, expression quantitative trait locus; lod, logarithm of odds ratio; QTL, quantitative trait locus; SD, sleep deprivation; TPF, theta-peak frequency

The EEG during REM sleep in the mouse is dominated by an almost single-frequency theta oscillation in the 5–9 Hz range of hippocampal origin [78], the main frequency of which can be easily determined with a Fourier transformation (Fig 6A). Theta activity during REM sleep is important for memory consolidation [79]. Our current data (see h^2^ analysis above) confirm our previous observations that most of the variance in TPF among inbred strains of mice can be explained by additive genetic factors [80,81]. Here, we discovered that increased sleep pressure shifts REM sleep TPF (compared to REM sleep TPF in corresponding baseline hours, i.e., ZT6–12) and that the direction of this shift strongly depends on genetic background (Fig 6A). TPF was a unique phenotype not part of any phenotypic module or supercluster (S2 Fig).

For this phenotype, we found a significant QTL on chromosome 4 (LOD = 4.94, 104–123 Mb; 50% variance explained) and a suggestive QTL chromosome 8 (LOD = 2.73, 0–15 Mb; 32% variance explained; Fig 6C top). The prioritization strategy identified *cytochrome P450*, *family 4*, *subfamily a*, *polypeptide 32* (*Cyp4a32*) as the top candidate gene in the liver (Fig 6C bottom). *Cyp4a32*, which was not expressed in cortex, is located within the associated chromosome 4 *ph*QTL region; is under strong *cis*-*e*QTL effect (*rs27480007*, chr4: 115.2 Mb, *q* = 1.0e−12), greatly increasing its expression in D2-allele carriers; and contains a nonsynonymous protein-damaging variation in the coding region of the D2 allele (V314E, PolyPhen2 score = 1.0, see Materials and methods). SD causes TPF to accelerate in carriers of the D2-allele at the *Cyp4a32 cis*-*e*QTL locus and to slow down in B6 carriers. *Cyp4a32* expression in D2-allele carriers is high in baseline and increases further after SD while remaining low and stable under both conditions in B6-allele carriers (Fig 6D). The 2 F1 hybrids both have a positive TPF shift, suggesting a dominance of the D2 allele, although some D2 allele–carrying lines did show a negative TPF shift (Fig 6F), indicating that this variation is not sufficient and possibly interacts with other loci, such as the suggestive QTL on chromosome 8, and with metabolites. Hiveplot visualization revealed that the TPF shift was associated with several amino acids (Fig 6B), which were all significantly down-regulated after SD (see above and Fig 4B). The 3 top-ranked associated amino acids were the branched-chain amino acids (BCAAs) leucine, isoleucine, and valine. Plasma levels of valine, in turn, were significantly linked to *Cyp4a32* expression (Fig 6E highlight), although no common *m*QTL was found.

*Cyp4a32* and its human ortholog *CYP4A11* are part of the *Cyp4a* gene family encoding cytochrome 450 liver enzymes that can ω-hydroxylate fatty acids and which are induced by starvation and diabetes [82]. *Cyp4a32* encodes a peptide targeting the degradation of arachidonic acid (ARA) specifically. ARA is abundant in the brain, but its levels largely depend on supply by blood [83]. ARA and its metabolites, such as prostaglandins and endocannabinoids, are involved in many processes in the brain—including signaling, synaptic plasticity, long-term potentiation, and neurogenesis—and have been associated in cognitive performance, mood, and neurodegenerative disease [83–85]. The relation between TPF and fatty acid metabolism has already been suggested with the identification of *Acads*, an acyl-CoA dehydrogenase involved not only in fatty-acid β-oxidation but also in BCAA degradation (Kyoto Encyclopedia of Genes and Genomes [KEGG]: mmu00280), as the causative gene explaining REM sleep TPF differences between 2 inbred strains [81] but not the SD-induced shift in TPF reported here. BCAAs, in turn, are involved in fatty acid biosynthesis [86,87] and are also implicated in insulin resistance [88]. These results suggest a pathway relating the SD effects on BCAA, and possibly ARA, through fatty acid metabolism in the periphery, with the marked SD-induced changes in TPF during REM sleep.

#### Example 4: Compensation for NREM sleep time lost

**Fig 7:**
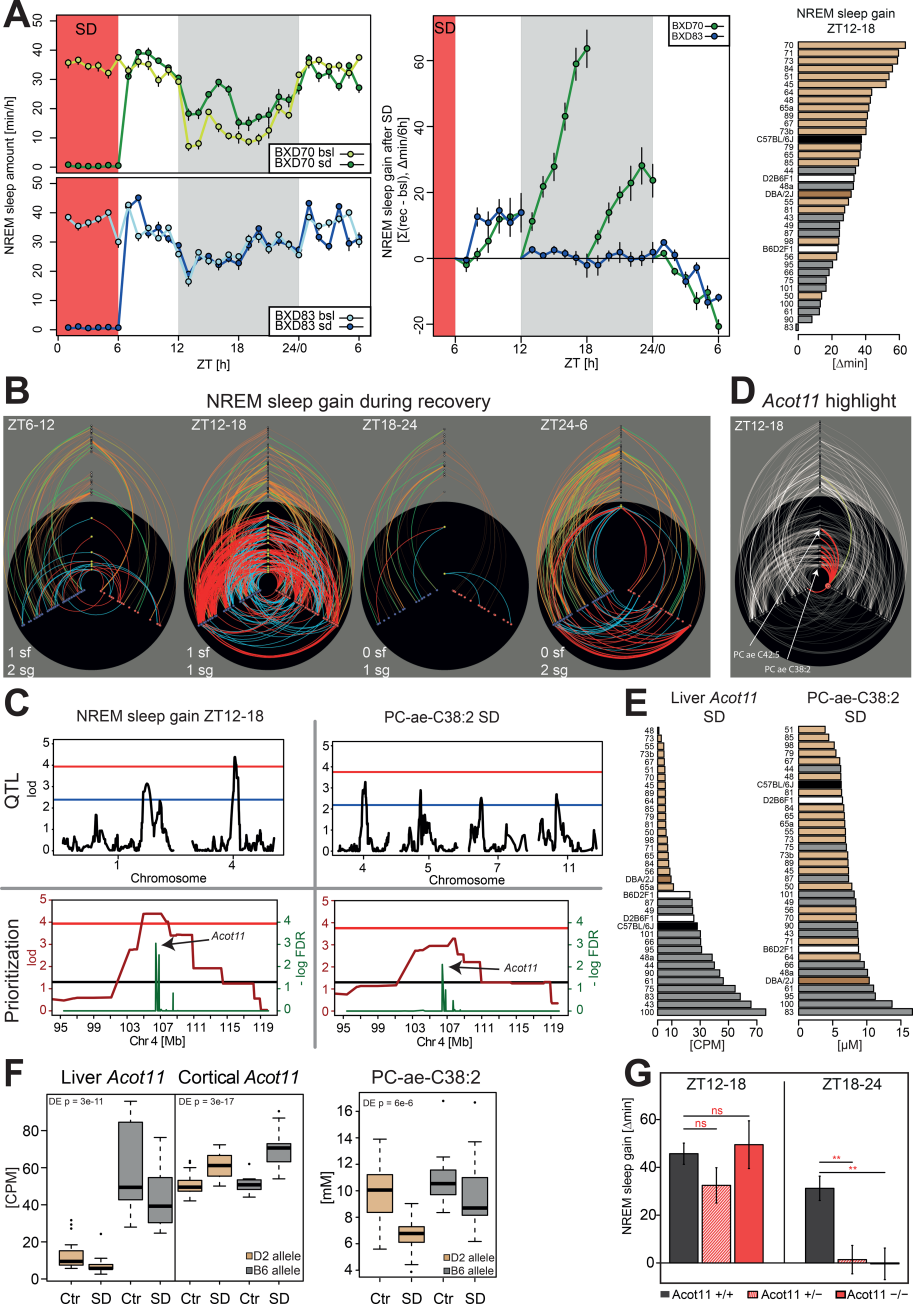
NREM sleep gain in the first 6 h of the dark period after SD is associated with *Acot11*. (A) Time course of hourly values of time spent in NREM sleep in baseline, SD (red area), and recovery for the 2 BXD lines showing the largest (BXD70; green) and lowest (BXD83; blue) NREM sleep gain during ZT12–18 (left). NREM sleep gain during 4 consecutive 6 h intervals during recovery compared to corresponding baseline intervals shows that in the recovery dark period (gray area), BXD83 mice did not accumulate extra NREM sleep, while BXD70 mice gained 88 min (middle). Strain distribution of ZT12–18 NREM sleep gain (right). B6-allele carriers compensated less for NREM sleep lost during SD than D2-allele carriers. For color-coding, see Fig 4. (B) Hiveplots for NREM sleep gain in 4 six-hour recovery intervals after the end of SD at ZT6. Compared to the other 3 intervals, NREM sleep gain was strongly associated with a number of metabolites during the second 6 h interval, i.e., ZT12–18. (C) NREM sleep gain during ZT12–18 mapped to a significant QTL on chromosome 4, explaining 45% of the total phenotypic variance (top left). PC-ae-C38:2 mapped suggestively to the same region (top right). Prioritization of liver transcripts for both phenotypes yielded *Acot11* as top-ranked, significant gene (bottom). (D) Hiveplot for the ZT12–18 NREM sleep gain, highlighting *Acot11*. *Acot11* was positively correlated with several phosphatidylcholines and to *Ovgp1* expression in the cortex. (E) Allelic effect of the chromosome 4–associated region on *Acot11* expression and PC-ae-C38:2 levels in the BXDs. *Acot11* expression in liver after SD was under a strong *e*QTL effect (*p* = 1.6e−13) with *B6-*allele carriers showing a higher *Acot11* expression than *D2*-allele carriers. *B6-*allele carriers also showed higher PC-ae-C38:2 levels after SD. (F) Both *Acot11* and PC-ae-C38:2 levels changed after SD. *Acot11* in liver and PC-ae-C38:2 in blood were significantly down-regulated. In the cortex, *Acot11* was, however, significantly up-regulated, and the chromosome 4–associated region did not modulate cortical *Acot11* expression. (G) Mice carrying 1 or 2 KO alleles for *Acot11* displayed less extra NREM sleep during recovery. In contrast to the BXD panel, this difference was present in the second (ZT18–24, right) and not during the first (ZT12–18, left panel) 6 h of the recovery dark period. *Acot11*, *acyl-CoA thioesterase 11*; CPM, counts per million; Ctr, control; *e*QTL, expression quantitative trait locus; KO, knockout; NREM, non-REM; PC-ae, phosphatidylcholine acyl-alkyl; QTL, quantitative trait locus; SD, sleep deprivation; ZT, zeitgeber time

During recovery sleep, mice compensate for the sleep lost during the preceding SD not only by sleeping deeper (quantified as the increase in EEG delta power discussed in Example 1) but also by sleeping more [89]. We quantified the gain in NREM and REM sleep time over the 24 h recovery period following the SD by contrasting these recovery values to time-matched baseline values within individual mice. We found that the gain for both NREM and REM sleep was largest in the first 6 h of the recovery dark period (ZT12–18; Fig 7A), consistent with our earlier observations [89]. However, only for the NREM sleep gain during that period did we identify a significant QTL on chromosome 4 (LOD = 4.38; 103–110 Mb), explaining 45% of the variance in this trait (Fig 7C). A second suggestive QTL was found on chromosome 1 (LOD = 3.14; 169–173 Mb; 35% variance explained). Together, the 2 loci explained 55% of the variance in NREM sleep gain (estimated using an additive model; see Materials and methods). Neither QTL was associated with the gain in REM sleep during this period (not even at the suggestive level), further underscoring the different regulation, both genetic and physiological, of these 2 sleep states.

NREM sleep gain during ZT12–18 clustered with the loss of time spent awake over the same time interval only (S2 Fig) and not with NREM sleep gain in the other 6 h intervals during the 24 h recovery. Hiveplot visualization of NREM sleep gain over the 24 h recovery period readily revealed the contrasting systems genetics “landscapes” for the 4 consecutive 6 h recovery intervals, with the ZT12–18 interval yielding far more connections at all 4 levels of analysis (Fig 7B). For instance, during this interval, 15 metabolites were highly correlated to NREM sleep gain, none of which were observed in the hiveplots of the other 6 h recovery intervals. All 15 metabolites were long phosphatidylcholines, and for 2 among those (PC-ae-C38:2 [LOD = 3.27; chr4: 101–110 Mb; 37% variance explained] and PC-ae-C42:5 [LOD = 3.02; chr4: 101–110 Mb; 31% variance explained]), suggestive *m*QTLs were identified, both mapping to the chromosome 4 *ph*QTL for NREM sleep gain (Fig 7C). Moreover, the ZT12–18 NREM sleep gain was correlated with the expression of 88 genes in cortex and 145 genes in liver that were all but 1 unique to this recovery interval. The genes with the highest number of connections to metabolites (≥10) were *Phf23*, *Rad54b*, and *Slc38a2* in the cortex and *Noc4l* and *Nat1* in liver.

Gene prioritization identified *acyl-CoA thioesterase 11* (*Acot11*) as the top candidate gene independently for the gain in NREM sleep and for PC-ae-C38:2 levels (Fig 7C), while for PC-ae-C42:5, no gene passed the prioritization FDR threshold. Nevertheless, both metabolites were significantly linked to *Acot11* expression, as can be seen in the hiveplot highlight for NREM sleep gain at ZT12–18, along with 6 other phosphatidylcholines (Fig 7D). A significant *cis*-*e*QTL was found that explained the differences in *Acot11* expression levels among BXD lines in liver after SD (*rs28135130*, chr4: 106.3Mb, *q* = 1e−13) but not in cortex. Liver *Acott11* expression in mice carrying the D2-allele at the *cis*-*e*QTL region was close to zero (Fig 7E and 7F). This near-zero expression in D2-allele carriers was even more pronounced for the shorter *Acot11* isoform (*NM_025590*), which was the more abundant isoform in the liver of B6-allele carriers (S5 Fig). By contrast, the D2-allele did not alter the expression of the short isoform in the cortex, and in both genotypes, its expression was higher than that of the less prevalent, longer isoform (*NM_001347159*; S5 Fig). Moreover, expression of the longer isoform was not affected by genotype. Besides the tissue- and isoform-specific regulation of *Acot11* expression, SD differentially modified *Acot11* expression in cortex and liver. The strong *cis*-*e*QTL effect associated with *Acot11* expression in the liver after SD was not present in the cortex for the control condition (*q* = 0.5) and only marginal after SD (*q* = 7e−4). *Acot11* was down-regulated in liver after SD but up-regulated in the cortex (Fig 7F).

D2-allele carriers display lower plasma PC-ae-C38:2 levels and have a larger NREM sleep gain during ZT12–18 (Fig 7E). While the majority of the BXD lines compensated by sleeping significantly more than baseline during ZT12–18 (+33.0 min on average), only BXD83 showed a negative gain (−1.3 min, Fig 7A). BXD83 is also the line with the highest PC-ae-C38:2 plasma levels and the third-highest *Acot11* expression in liver after SD (Fig 7E). It is intriguing that the NREM sleep gain and PC-ae-C38:2 levels measured in the parental strains are closer to that in BXD lines carrying the opposite allele (Fig 7A and 7E). This reinforces the idea that these phenotypes are due to multiple gene × gene interactions. It should be kept in mind that in these analyses, the metabolome and transcriptome data were obtained in tissues collected immediately after the SD (ZT6), while the gain in NREM sleep time was quantified in the ensuing recovery. Thus, changes in *Acot11* expression and/or PC-ae-C38:2 levels seem to predispose to differences in NREM sleep recovery occurring later.

*Acot11* is an acyl-CoA thioesterase that catalyzes the hydrolysis of long fatty acyl-CoAs to form FFAs and is therefore important in the homeostatic regulation and turnover of FFAs [90]. *Acot11*-knockout mice show increased energy expenditure and are resistant to diet-induced obesity and its metabolic consequences [91]. We used these *Acot11*-knockout mice to verify the causal involvement of *Acot11* in NREM sleep gain in mice. In the line used, the knockout allele was brought onto a B6 background through repeated (>20) backcrossing. Both heterozygous and homozygous null allele carriers were deficient in NREM sleep gain compared to their wild-type littermate controls (Fig 7G), confirming that *Acot11* is causally implicated in NREM sleep recovery. The difference in NREM sleep gain occurred, however, in the second half (ZT18–24) and not, as was the case in the BXD panel, in the first half (ZT12–18) of the recovery dark period.

In humans, SD induces an increase in circulating FFAs [92]. Because both elevated plasma FFA levels [93,94] and sleep restriction [95,96] can lead to insulin resistance and predispose to metabolic disease, including type 2 diabetes, Broussard and colleagues proposed that the effects of sleep restriction on FFA levels might present a mechanism by which sleep restriction causes insulin resistance and increased type 2 diabetes risk [92]. Our data implicate *Acot11* as a molecular player in this mechanistic link between sleep restriction and its adverse effects on fatty acid metabolism.

## Discussion

We have generated a rich, multidimensional, experimentally determined knowledge base, drawing on 4 levels of organization from the DNA level to steady-state RNA levels in brain and liver, circulating metabolites, and a deep phenome of sleep-wake-related phenotypes, all under 2 experimental conditions. At the core of this knowledge base is the BXD ARIL resource. This mouse GRP provides a “population model” with a controlled and stable degree of genetic variation, each line carrying a fixed and unique pattern of recombination of the 2 parental chromosomes [17]. The panel segregates for approximately 5.2 million sequence variants corresponding to about half of all common genetic variation among classic laboratory mouse strains [97]. This level of genetic complexity exceeds that in many human populations, such as the Icelandic and Finnish populations that have been so useful in genetics of disease [98–100]. Our results underscore the power of the BXD panel in discovering the genetic and molecular underpinnings of clinically relevant traits already demonstrated in other research fields [19–21].

We extracted 341 sleep-wake-related phenotypes belonging to 120 distinct phenotypic modules from each individual mouse. Half of these phenotypes had higher than 0.68 heritability, indicating that they are amenable to genetic dissection even when using only 33 ARILs. Although numerous knockout studies have shown that (lack of) single genes impact many of the phenotypes we quantified (for review, see [8,101]), we demonstrate here that even highly heritable traits are determined by the interaction of several small-effect loci. Two striking examples of such traits are TPF during REM sleep and the gain in δ2 power after SD, for which we identified 4 and 5 suggestive QTLs, respectively, that together explained 58% and 75% of the genetic variance in these 2 traits. Thus, while reductionist approaches have been successful at identifying genes affecting sleep in a mendelian fashion, when studied at a more natural population level, most of these phenotypes represent complex traits, and mendelian (or null) alleles are likely to play a lesser role. To systematically explore these nonadditive, multiloci interactions at the level of the whole genome, innovative algorithms in the area of machine learning are needed. Currently, more than 2-way epistatic interactions are computationally challenging. We are therefore now exploring novel multiloci epistatic approaches to extract this type of information (see, e.g., [102,103]).

With the 4 examples described, we could only illustrate a fraction of all the novel information contained in our experimentally derived knowledge base. Here, we focused on the effects of sleep loss exclusively because systems genetics resources in this research domain are lacking and because of the immediate clinical relevance of these effects. Importantly, the pathways we identified were unique to the sleep-deprivation condition and did not explain phenotypic variance of the respective traits under undisturbed baseline conditions. This illustrates that already a relatively mild sleep disruption (preventing sleep during half of the rest phase) extensively reshapes the systems genetics landscape.

The power of systems genetics lies in generating hypotheses. In the current dataset, several observations imply SD to challenge fatty acid turnover. Besides *Acot11*—which regulates the levels of FFAs and, as we show here, the recovery of NREM sleep—also *Cyp4a32*, which contributes to the SD-induced shift in the frequency of theta oscillation in REM sleep, encodes an enzyme regulating fatty acid levels. This frequency shift was strongly correlated with levels of the branched amino acids leucine, isoleucine, and valine, which, in turn, are part of a fatty acid biosynthesis pathway. The link between *Cyp4a32* and the dominant frequency of theta oscillatory activity also illustrates the importance of a peripheral molecular pathway in regulating brain activity, as *Cyp4a32* was not expressed in brain. This finding is of relevance because although many studies have emphasized the deleterious effect of sleep loss on peripheral systems, research on the substrate of sleep need largely remains brain centric. In addition, *Pla2g4e* and *Mlycd*, the 2 genes with the strongest *cis-e*QTL effect for their DE after SD, both encode enzymes affecting fatty acid metabolism. *Acot11*, the *Cyp4a* gene family, FFA levels, and sleep restriction have all been linked to obesity and insulin resistance [82,91,93–96]. Another pathway of importance in mediating the effects of sleep loss concerns AMPA-R trafficking supported by the 8-fold increase in cortical *Arc* expression and *Kif16b*’s role in shaping δ2 power after SD. Both genes encode proteins involved in the endosomal trafficking of AMPA-Rs (see Results) that have already been explored as therapeutic targets to counter the deleterious effects of SD on cognition [73,76]. Finally, *Wrn*‘s association with EEG slow waves during NREM sleep offers a model system to mechanistically study the molecular pathways underlying the characteristic age-related decrease in the prevalence of EEG slow waves and sleep quality.

Hypotheses concerning the involvement of the pathways in the sleep homeostatic process we discovered need to be further tested experimentally. With a reverse genetics approach, we could already confirm *Acot11*’s role in the recovery of sleep time lost. This approach is, however, not always informative or possible, because a lack of protein on a given genetic background is unlikely to mimic the impact of an allelic variant in a genetically diverse population, or the knockout might be lethal, as is the case for *Kif16b* [69]. Efforts to comprehensively phenotype (including sleep) knockouts for all known and predicted mouse genes by the International Mouse Phenotyping Consortium (IMPC; www.mousephenotype.org) are ongoing, but unfortunately, no knockouts for the 4 genes we highlight here have been submitted for phenotyping. Another important community resource is the mostly mouse-oriented database GeneNetwork (www.genenetwork.org), which hosts a massive amount of phenotypic and molecular information collected by the many researchers using the same BXD resource. We are in the process of structuring our database to enable sharing of the integrated data in GeneNetwork according to the FAIR data management concepts [104]. Furthermore, cross-species validation in, e.g., humans, flies, and *Caenorhabditis elegans* and Genome-Wide Association Study (GWAS) and biobank database searches are important additional ways of validating and extending our mouse observations. According to the human GWAS databases grasp.nhlbi.nih.gov and www.ebi.ac.uk/gwas/, SNP variants in *Acot11* are significantly associated with (among others) the rate of cognitive decline in Alzheimer disease, behavioral disinhibition, cardiovascular disease, and triglyceride levels. Variants in *Wrn* are associated with aging and time to death, cardiovascular disease, cholesterol, and daytime rest. Finally, variants in the human ortholog of *Cyp4a32*, *CYP4A11*, are associated with blood metabolite levels, including amino acids and acyl carnitines, and *Kif16b* variants with intelligence.

A first evaluation of the systems genetics field has highlighted a clear need for better communication, “open science,” and collaboration among groups [24]. Toward this aim, we have shared our results and analyses through an easily accessible and reproducibility-oriented web interface that accompanies this publication. We hope that the interactivity of the web interface will encourage the reader to further mine our data, thereby reproducing our conclusions and, hopefully, discovering other key regulators and pathways. In our analyses, we have also strived to follow the concepts of the FAIR data management approach [104], resulting in a data life cycle management plan, open access provided by the web interface for data mining, and, importantly, interoperability. The implementation of the FAIR approach will be illustrated in an accompanying publication.

In summary, we have applied a systems genetics approach to uncover new genes and pathways associated with the effects of sleep loss, an approach thought critical for predicting disease susceptibility [18]. This integrative, multilevel approach allowed us to follow the flow of information from DNA variants to molecular intermediate phenotypes to behavioral and electrophysiological end phenotypes, and to assess how this network of multiscale effects is perturbed by an environmental challenge. The information gained could not have been achieved through other genetic approaches that are based on the “1-gene-to-1-phenotype” approach. Moreover, with the tools and web interface we developed, our open-access knowledge base provides a unique resource that goes well beyond merely cataloguing and ranking *ph-*, *m-*, and *e*QTLs. Furthermore, owing to the use of a GRP, the database and its content are easily scalable. A first challenge will be to complement the dataset with females of the same lines. In addition, we are expanding the database with an additional intermediate phenotype—namely, the SD-induced changes in chromatin accessibility—aiming to identify the variants in noncoding regulatory elements that could predict the varying molecular and phenotypic response to sleep loss. Proteome, microbiome, and inflammasome data are obvious other intermediate phenotypes that will further strengthen this knowledge base and increase its value to, e.g., assist with identifying biomarkers gauging sleep pressure and potential therapeutic targets for sleep-wake-related disorders.

## Materials and methods

### Ethics statement

All experiments followed international guidelines and were approved by the veterinary authorities of the state of Vaud, Switzerland (SCAV authorization #2534). Animals assigned to Experiment 1 (see Experimental design below and Fig 1) were equipped with chronic EEG and EMG electrodes under deep anesthesia according to methods described in detail in [105]. In short, IP injection of Xylazine (10 mg/kg)/Ketamine (100 mg/kg) ensures a deep plane of anesthesia for the duration of the surgery (i.e., around 30 min). Analgesia was provided the evening prior and the 3 d after surgery with Dafalgan in the drinking water (200–300 mg/kg). Mice were allowed to recover for at least 10 d prior to baseline recordings. Animals assigned to Experiment 2 (see Experimental design below and Fig 1) were killed by decapitation after being anesthetized with isoflurane, upon which blood, cerebral cortex, and liver samples were collected immediately.

### Animals, breeding, and housing conditions

We phenotyped 33 BXD RI strains originating from the University of Tennessee Health Science Center (Memphis, TN, United States of America). The 33 lines were randomly chosen from the then available, newly generated ARIL panel [17], although lines with documented poor breeding performance were not considered. Two breeding trios per BXD strain were purchased from a local facility (EPFL-SV, Lausanne, Switzerland) and bred in-house until sufficient offspring was obtained. The parental strains D2 and B6 and their reciprocal F1 offspring (B6D2F1 [BD-F1] and D2B6F1 [DB-F1]) were bred and phenotyped alongside. Suitable (age and sex) offspring was transferred to our sleep-recording facility, where they were singly housed, with food and water available ad libitum, at a constant temperature of 25°C and under a 12 h light/12 h dark cycle (LD12:12, fluorescent lights, intensity 6.6 cds/m^2^, with ZT0 and ZT12 designating light and dark onset, respectively). Male mice aged 11–14 wk at the time of experiment were used for phenotyping, with a mean of 12 animals per BXD line among all experiments. Note that 3 BXD lines had a lower replicate number (*n*), with respectively BXD79 (*n* = 6), BXD85 (*n* = 5), and BXD101 (*n* = 4) because of poor breeding success. For the remaining 30 BXD lines, replicates were distributed as follows: for EEG/behavioral phenotyping (Experiment 1 in Fig 1; mean = 6.2/line; 5 ≤ *n* ≤ 7) and for molecular phenotyping (Experiment 2 in Fig 1; mean = 6.8/line; 6 ≤ *n* ≤ 9). Additionally, to assess the stability of outcome variables over time, parental lines were phenotyped twice—i.e., at the start (labeled B6-1 and D2-1) and end (labeled B6-2 and D2-2) of the breeding and data-collecting phase, which spanned 2 y (March 2012–December 2013). To summarize, distributed over 32 experimental cohorts, 227 individual mice were used for behavioral/EEG phenotyping (Experiment 1) and 256 mice for tissue collection for transcriptome and metabolome analyses (Experiment 2), the latter being divided into sleep deprived (SD) and controls (“Ctr”; see Experimental design section below). We strived to randomize the lines across the experimental cohorts so that biological replicates of 1 line were collected/recorded on more than 1 occasion while also ensuring that an even number of mice per line was included for tissue collection so as to pair SD and “Ctr” individuals within each cohort (for behavioral/EEG phenotyping, each mouse serves as its own control).

### Experimental design

The study consisted of 2 experiments, i.e., Experiments 1 and 2 (Fig 1). Animals of both experiments were maintained under the same housing conditions. Animals in Experiment 1 underwent surgery and, after a >10 d recovery period, EEG and LMA were recorded continuously for a 4 d period starting at ZT0. The first 2 d were considered baseline (B1 and B2). The first 6 h of Day 3 (ZT0–6), animals were sleep deprived in their home cage by “gentle handling” [105]. The remaining 18 h of Day 3 and Day 4 were considered recovery (R1 and R2). Half of the animals included in Experiment 2 were sleep deprived (SD) alongside the animals of Experiment 1. The other half was left undisturbed in another room (i.e., control or Ctr). Both SD and “Ctr” mice of Experiment 2 were killed at ZT6 (i.e., immediately after the end of the SD) for sampling of liver and cerebral cortex tissue as well as trunk blood. All mice were left undisturbed for at least 2 d prior to SD.

#### Experiment 1: EEG/EMG and LMA recording and analysis

EEG/EMG surgery was performed under deep anesthesia according to our standard methods [105]. EEG and EMG signals were amplified, filtered, digitized, and stored using EMBLA (Medcare Flaga, Thornton, CO, USA) hardware (A10 recorder) and software (Somnologica). LMA was recorded by passive infrared (PIR) sensors (Visonic, Tel Aviv, Israel) at 1 min resolution for the duration of the 4 d experiment, using ClockLab (ActiMetrics, IL, USA).

Offline, the sleep-wake states wakefulness, REM sleep, and NREM sleep were annotated on consecutive 4 s epochs, based on the EEG and EMG patterns. To assist the annotation of this extensive dataset (around 20 million 4 s epochs), we developed a semiautomated scoring system. The 4 d recordings of 43 mice (19% of all recordings), representing animals from 12 strains, were fully annotated visually by an expert according to established criteria [105]. Due to large between-line variability in EEG signals, even after normalization, a partial overlap of the different sleep-wake states remained, as evidenced by the absolute position of the center of each state cluster, which differed even among individuals of the same line (precluding the use of 1 “reference” mouse), even per line, to reliably annotate sleep-wake states for the others (S1 Fig). To overcome this problem, 1 d out of 4 (i.e., Day 3 or R1, which includes the SD) was visually annotated for each mouse. These 4 s sleep-wake scores were used to train the semiautomatic scoring algorithm, which took as input 82 numerical variables derived from the analyses of EEG and EMG signals using frequency- (discrete Fourier transform [DFT]) and time-domain analyses performed at 1 s resolution. We then used these data to train a series of support vector machines (SVMs) [106] specifically tailored for each mouse, using combinations of the 5 or 6 most informative variables out of the 82 input variables. The best-performing SVMs for a given mouse were then selected based on the upper-quartile performance for global classification accuracy and sensitivity for REM sleep (the sleep-wake state with the lowest prevalence) and used to predict sleep-wake states in the remaining 3 d of the recording. The predictions for 4 consecutive 1 s epochs were converted into 1 four-second epoch. Next, the results of the distinct SVMs were collapsed into a consensus prediction, using a majority vote. In case of ties, epochs were annotated according to the consensus prediction of their neighboring epochs. A representative example of prediction is shown in S1 Fig. To prevent overfitting and assess the expected performance of the predictor, only 50% of the R1 manually annotated data from each mouse were used for training. The classification performance was assessed by comparing the automatic and visual scoring of the fully manually annotated 4 d recordings of 43 mice. The global accuracy was computed using a confusion matrix [107] of the completely predicted days (B1, B2, and R2; S1 Fig). For all subsequent analyses, the visually annotated Day 3 (R1) recording and the algorithmically annotated days (B1, B2, and R2) were used for all mice, including those for which these days were visually annotated.

We quantified 341 phenotypes based on the sleep-wake states, LMA, and the EEG signal, constituting 3 broad phenotypic categories. The 96 h sleep-wake sequence of each animal was used to directly assess traits in 3 “state”-related phenotypic subcategories: (i) duration (e.g., time spent in wakefulness, NREM sleep, and REM sleep, both absolute and relative to each other, such as the ratio of time spent in REM versus NREM); (ii) aspects of their distribution over the 24 h cycle (e.g., time course of hourly values, midpoint of the 12 h interval with highest time spent awake, and differences between the light and dark periods); and (iii) sleep-wake architecture (e.g., number and duration of sleep-wake bouts, sleep fragmentation, and sleep-wake state transition probabilities). Similarly, overall activity counts per day, as well as per unit of time spent awake, and the distribution of activity over the 24 h cycle were extracted from the LMA data. EEG signals of the 4 different sleep-wake states (wakefulness, NREM sleep, REM sleep, and theta-dominated waking [TDW], see below) were quantified within the 4 s epochs matching the sleep-wake states using DFT (0.25 Hz resolution, range 0.75–90 Hz, window function Hamming). Signal power was calculated in discrete EEG frequency bands—i.e., delta (1.0–4.25 Hz, δ), slow delta (1.0–2.25 Hz; δ1), fast delta (2.5–4.25; δ2), theta (5.0–9.0 Hz; θ), sigma (11–16 Hz; σ), beta (18–30 Hz; β), slow gamma (32–55 Hz; γ1), and fast gamma (55–80 Hz; γ2). Power in each frequency band was referenced to total EEG power over all frequencies (0.75–90 Hz) and all sleep-wake states in days B1 and B2 to account for interindividual variability in absolute power. The contribution of each sleep-wake state to this reference was weighted such that, e.g., animals spending more time in NREM sleep (during which total EEG power is higher) do not have a higher reference as a result [80]. Moreover, the frequency of dominant EEG rhythms was extracted as phenotypes, specifically that of the theta rhythm characteristic of REM sleep and TDW. The latter state, a substate of wakefulness, defined by the prevalence of theta activity (6.0–10.0 Hz) in the EEG during waking [78,108], was quantified according to the algorithm described in [46]. We assessed the time spent in this state, the fraction of total wakefulness it represents, and its distribution over 24 h. Finally, discrete, paroxysmal events were counted, such as sporadic spontaneous seizures and neocortical spindling, which are known features of D2 mice [109], which we also found in some BXD lines.

All phenotypes were quantified in baseline and recovery separately, and the effect of SD on all variables was computed as recovery versus baseline differences or ratios. The recovery-to-baseline contrasts are the focus of this paper. Obviously, some of the 341 phenotypes are strongly correlated (e.g., the time spent awake and asleep in a given recording interval), resulting in identical QTLs (albeit with different association strengths). To estimate the number of unique phenotypes, we clustered highly correlated phenotypes into modules. We then counted the number of phenotype categories and subcategories within each module (S2 Fig). We obtained 120 modules or 148 when considering phenotypes of different subclasses (e.g., “EEG,” “State,” or “LMA”) within a module as separate. Please see the “Swiss-BXD” web interface (https://bxd.vital-it.ch; Downloads, General_Information.xlsx) for a full listing of all phenotypes quantified and the modules they were part of.

#### Experiment 2: Tissue collection and preparation

Mice were killed by decapitation after being anesthetized with isoflurane, and blood, cerebral cortex, and liver were collected immediately. The whole procedure took no more than 5 min per mouse. Blood was collected at the decapitation site into tubes containing 10 ml heparin (2 U/μl) and centrifuged at 4,000 rpm during 5 min at 4°C. Plasma was collected by pipetting, flash-frozen in liquid nitrogen, and stored at −80°C until further use. Cortex and liver were flash-frozen in liquid nitrogen immediately after dissection and were stored at −140°C until further use.

For RNA extraction, frozen samples were homogenized for 45 s in 1 ml of QIAzol Lysis Reagent (Qiagen; Hilden, Germany) in a gentleMACS M tube using the gentleMACS Dissociator (Miltenyi Biotec; Bergisch Gladbach, Germany). Homogenates were stored at −80°C until RNA extraction. Total RNA was isolated and purified from cortex using the automated nucleic acid extraction system QIAcube (Qiagen; Hilden, Germany) with the RNeasy Plus Universal Tissue mini kit (Qiagen; Hilden, Germany) and were treated with DNAse. Total RNA from liver was isolated and purified manually using the Qiagen RNeasy Plus mini kit (Qiagen; Hilden, Germany), which includes a step for effective elimination of genomic DNA. RNA quantity, quality, and integrity were assessed utilizing the NanoDrop ND-1000 spectrophotometer (Thermo scientific; Waltham, Massachusetts, USA) and the Fragment Analyzer (Advanced Analytical). The 256 mice initially killed for tissue collection yielded 222 cortex and 222 liver samples of good quality.

Equal amounts of RNA from biological replicates (3 samples per strain, tissue, and experimental condition, except for BXD79, BXD85, and BXD101; see above under Animals, breeding, and housing conditions) were pooled, yielding 156 samples for library preparation. RNA-seq libraries were prepared from 500 ng of pooled RNA using the Illumina TruSeq Stranded mRNA reagents (Illumina; San Diego, California, USA) on a Caliper Sciclone liquid handling robot (PerkinElmer; Waltham, Massachusetts, USA). Libraries were sequenced on the Illumina HiSeq 2500 using HiSeq SBS Kit v3 reagents, with cluster generation using the Illumina HiSeq PE Cluster Kit v3 reagents. A mean of 41 M 100 bp single-end reads were obtained (29 M ≤ *n* ≤ 63 M).

Targeted metabolomics analysis was performed using flow injection analysis (FIA) and liquid chromatography/mass spectrometry (LC/MS) as described in [36,110]. To identify metabolites and measure their concentrations, plasma samples were analyzed using the AbsoluteIDQ p180 targeted metabolomics kit (Biocrates Life Sciences AG, Innsbruck, Austria) and a Waters Xevo TQ-S mass spectrometer coupled to an Acquity UPLC liquid chromatography system (Waters Corporation, Milford, MA, USA). The kit provided absolute concentrations for 188 endogenous compounds from 6 different classes, namely acyl carnitines, amino acids, biogenic amines, hexoses, glycerophospholipids, and sphingolipids. Plasma samples were prepared according to the manufacturer’s instructions. Sample order was randomized, and 3 levels of quality controls (QCs) were run on each 96-well plate. Data were normalized between batches, using the results of quality control level 2 (QC2) repeats across the plate (*n* = 4) and between plates (*n* = 4) using Biocrates METIDQ software (QC2 correction). Metabolites below the lower limit of quantification or the limit of detection, as well as above the upper limit of quantification, or with standards out of limits, were discarded from the analysis [110]. Out of the 188 metabolites assayed, 124 passed these criteria across samples and were used in subsequent analyses. No hexoses were present among the 124 metabolites. Out of the 256 mice killed for tissue collection, 249 plasma samples were used for this analysis. An average of 3.5 animals (3 ≤ *n* ≤ 6) per line and experimental condition were used (except for BXD79, BXD85, and BXD101 with respectively 2, 1, and 1 animal/condition used; see above under Animals, breeding, and housing conditions). Note that in contrast to the RNA-seq experiment, samples were not pooled but analyzed individually.

In the same plasma samples, we determined corticosterone levels using an enzyme immunoassay (corticosterone EIA kit; Enzo Life Sciences, Lausen, Switzerland) according to the manufacturer’s instructions. All samples were diluted 40 times in the provided buffer, kept on ice during the manipulation, and tested in duplicate. BXD lines were spread over multiple 96-well plates in an attempt to control for possible batch effects. In addition, a “control” sample was prepared by pooling plasma from 5 C57BL/6 mice. Aliquots of this control were measured along with each plate to assess plate-to-plate variability. The concentration was calculated in pg/ml based on the average net optical density (at λ = 405 nm) for each standard and sample.

### RNA-seq analyses

RNA-seq data were processed using the Illumina Pipeline Software version 1.82. All RNA-seq samples passed FastQC quality thresholds (version 0.10.1) and could thus be used in subsequent analysis. For gene expression quantification, we used a standard pipeline that was already applied in a previous study [111]. Reads were mapped to MGSCv37/mm9 using the STAR splice aligner with the *2pass* pipeline [112]. Count data was generated using htseq-count from the HTseq package using parameters “stranded = reverse” and “mode = union” [113]. Gene boundaries were extracted from the mm9/refseq/reflat dataset of the UCSC table browser. EdgeR was then used to normalize read counts by library size. Genes with a mean raw read count below 10 were excluded from the analysis, and the raw read counts were normalized using the TMM normalization [114] and converted to log counts per million (CPM). Although for both tissues, the RNA-seq samples passed all quality thresholds, and among-strain variability was small, more reads were mapped in cortex than in liver (S6 Fig), and we observed a somewhat higher coefficient of variation in the raw gene read count in liver than in cortex (S6 Fig). To assess the DE between the sleep-deprived and control conditions, we used the R package limma [115] with the voom weighting function followed by the limma empirical Bayes method [116]. RNA-seq data are deposited in NCBI GEO (accession code GSE114845).

The RNA-seq dataset was also used to complement the publicly available GeneNetwork genetic map (www.genenetwork.org), thus increasing its resolution. RNA-seq variant calling was performed using the Genome Analysis ToolKit (GATK) from the Broad Institute, using the recommended workflow for RNA-seq data [117]. To improve coverage depth, 2 additional RNA-seq datasets from other projects using the same BXD lines were added [111]. In total, 6 BXD datasets from 4 different tissues (cortex, hypothalamus, brainstem, and liver) were used. A hard filtering procedure was applied as suggested by the GATK pipeline [117–119]. Furthermore, genotypes with more than 10% missing information, low quality (<5,000), and redundant information were removed. GeneNetwork genotypes, which were discrepant with our RNA-seq experiment, were tagged as “unknown” (mean of 1% of the GeneNetwork genotypes/strain [0.05% ≤ *n* ≤ 8%]). Finally, GeneNetwork and our RNA-seq genotypes were merged into a unique set of around 11,000 genotypes, which was used for all subsequent analyses. This set of genotypes was already used successfully in a previous study of BXD lines [111] and is available through our “Swiss-BXD” web interface (https://bxd.vital-it.ch; Downloads, Genotypes.GeneNetwork2005AndRNAseq.geno).

Although overall, a close to 50/50 balance between B6 and D2 genotypes was observed across the genome, a minority of sites displayed a strong imbalance toward either genotype (S7 Fig). We also confirmed a minor but general trend toward more D2 than B6 genotypes per strain (S7 Fig), which was also found in the GeneNetwork genotypes for the BXD strains used in our study.

### QTL mapping

The R package *qtl/r* [120] was used for interval mapping of behavioral/EEG phenotypes (*ph*QTLs) and metabolites (*m*QTLs). Pseudomarkers were imputed every cM, and genome-wide associations were calculated using the Expected-Maximization (EM) algorithm. *p*-values were corrected for FDR using permutation tests with 1,000 random shuffles. The significance threshold was set to 0.05 FDR, a suggestive threshold to 0.63 FDR, and a highly suggestive threshold to 0.10 FDR according to [28,29]. QTL boundaries were determined using a 1.5 LOD support interval. To preserve sensitivity in QTL detection, we did not apply further *p*-value correction for the many phenotypes tested. Effect size of single QTLs was estimated using 2 methods. Method 1 does not consider eventual other QTLs present and computes effect size according to 1 − 10^(−(2/n)*LOD). Method 2 does consider multi-QTL effects and computes effect size by each contributing QTL by calculating first the full, additive model for all QTLs identified and, subsequently, estimating the effects of each contributing QTL by computing the variance lost when removing that QTL from the full model (“drop-one-term” analysis). For Method 2, the additive effect of multiple suggestive, highly suggestive, and significant QTLs was calculated using the *fitqtl* function of the *qtl/r* package [121]. With this method, the sum of single QTL effect estimation can be lower than the full model because of association between genotypes. In the Results section, Method 1 was used to estimate effect size, unless specified otherwise. It is important to note that the effect size estimated for a QTL represents the variance explained of the genetic portion of the variance (between-strain variability) quantified as heritability and not of the total variance observed for a given phenotype (i.e., within- plus between-strain variability).

For detection of *e*QTLs, *cis-e*QTLs were mapped using FastQTL [122] within a 2 Mb window for which adjusted *p*-values were computed with 1,000 permutations and beta distribution fitting. The R package *qvalue* [123] was then used for multiple-testing correction as proposed by [122]. Only the *q*-values are reported for each *cis-e*QTL in the text. *Trans-e*QTL detection was performed using a modified version of FastEpistasis [124], on several million associations (approximately 15,000 genes × 11,000 markers), applying a global, hard *p*-value threshold of 1E−4.

### Protein damage prediction

Variants detected by our RNA-seq variant calling were annotated using Annovar [125] with the RefSeq annotation dataset. Nonsynonymous variations were further investigated for protein disruption using Polyphen-2 version 2.2.2 [126], which was adapted for use in the mouse according to recommended configuration.

### Hiveplot visualization

Hiveplots were constructed with the R package *HiveR* [25] for each phenotype. Gene expression and metabolite levels represented in the hiveplots come from either the “Ctr” (control) or SD molecular datasets according to the phenotype represented in the hiveplot; i.e., the “Ctr” dataset is represented for phenotypes related to the baseline (“bsl”) condition, while the SD dataset is shown for phenotypes related to recovery (“rec” and “rec/bsl”). For a given hiveplot, only those genes and metabolites were included (depicted as nodes on the axes) for which the Pearson correlation coefficient between the phenotype concerned and the molecule passed a data-driven threshold set to the top 0.5% of all absolute correlations between all phenotypes on the one hand and all molecular (gene expression and metabolites) on the other. This threshold was calculated separately for “Bsl” phenotypes and for “Rec” and “Rec/Bsl” phenotypes and amounted to absolute correlation thresholds of 0.510 and 0.485, respectively. The latter was used for the recovery phenotypes in Results Examples 1–4 and for the printed hiveplots (other thresholds can be chosen in the interactive website https://bxd.vital-it.ch). Cross-associations between genes and metabolites represented by the edges in the hiveplot were filtered using quantile thresholds (top 0.05% gene–gene associations, top 0.5% gene–metabolite associations). We corrected for *cis*-*e*QTL confounding effects by computing partial correlations between all possible pairs of genes (see Results and Fig 4B and 4C for details).

### Candidate-gene prioritization strategy

In order to prioritize genes in identified QTL regions, we chose to combine the results of the following analyses: (i) QTL mapping (*ph*QTL or *m*QTL, Fig 2C), (ii) correlation analysis, (iii) expression QTL (*e*QTL, Fig 2B), (iv) protein damaging–variation prediction, and (v) DE (Fig 3A). Each result was transformed into an “analysis score” using a min/max normalization, in which the contribution of extreme values was reduced by a winsorization of the results (S4 Fig). These analysis scores were first associated with each gene (see below) and then integrated into a single "integrated score" computed separately for each tissue, yielding 1 integrated score in cortex and 1 in liver. The correlation analysis score, *e*QTL score, DE score, and protein damaging–variation score are already associated to genes, and these values were therefore simply attributed to the corresponding gene. To associate a gene with the *ph*-/*m*QTL analysis score (which is associated to markers), we used the central position of the gene to infer the associated *ph*-/*m*QTL analysis score at that position. In case of a *cis*-*e*QTL linked to a gene or a damaging variation within the gene, we used the position of the associated marker instead (S4 Fig). To emphasize diversity and reduce analysis score information redundancy, we weighted each analysis score using the Henikoff algorithm. The individual scores were discretized before using the Henikoff algorithm, which was applied on all the genes within the *ph*-/*m*QTL region associated with each phenotype (S4 Fig). The integrated score (formula in Fig 4D) was calculated separately for cortex and liver. We performed a 10,000-permutation procedure to compute an FDR for the integrated scores. For each permutation procedure, all 5 analysis scores were permutated, and a novel integrated score was computed again. The maximal integrated score for each permutation procedure was kept, and a significance threshold was set at quantile 95. Applying the Henikoff weighting improved the sensitivity of the gene prioritization. E.g., among the 91 behavioral/EEG phenotypes quantified with 1 or more suggestive/significant QTL after SD, 40 had at least 1 gene significantly prioritized with Henikoff weighting, against 32 without. Examples of analysis scores and weight can be found in S1 Table.

## Supporting information

S1 Fig EEG/EMG semiautomatic scoring. (A) Comparison of the normalized signal for 2 individual mice (top and bottom rows) of 2 BXD lines (left and right) and 1 parental line (DBA/2J; Middle), visually annotated by an expert scorer. Plotted are the peak-to-peak EMG amplitude (y-axis) against EEG delta (1.0–4.0 Hz) power (x-axis). (B) Example of predicted sleep-wake states of a representative 28 min section (420 four-s epochs) of mouse BXD045-1. Top row: state manually assigned by the expert. Second row: consensus of the automated prediction. Third row: results obtained for 11 distinct SVM predictors from which the consensus prediction is derived. (C) Accuracy values of the prediction for the 43 mice for which the 4 d recordings were fully annotated by the expert. The SVMs were trained on the R1 recording and then used to predict sleep-wake state for days B1, B2, and R2. Predicted sleep-wake states were compared to manual annotation using a confusion matrix (see Materials and methods). EEG, electroencephalography; EMG, electromyography; SVM, support vector machine
(TIF)

S2 Fig Relatedness among EEG and behavioral phenotypes. To quantify the relationship among phenotypes and to identify unique phenotype modules, we cross-correlated all 341 phenotypes using Spearman correlations followed by hierarchical clustering (average linkage). The resulting dendrogram was cut at a height of 0.3, thereby defining 120 modules. Phenotypes belonging to the same module but not to the same (sub-) category were counted separately, yielding 148 distinct phenotypic modules. The modules are represented by node color, and phenotype categories by node shape (see Fig 2A and Materials and methods). Edges were filtered for top correlation (|s| ≥ 0.7). Three “superclusters” (Supercluster I–III) grouping several modules were observed. The 4 recovery phenotypes discussed in Results section (Examples 1–4) are marked. EEG, electroencephalography.
(TIF)

S3 Fig BXD web application. All data presented are available in our web application: https://bxd.vital-it.ch. Examples and a tutorial can be found on the website. (A) Options to search genes and metabolites in either cortex or liver, with Pearson correlation thresholds selection. (B) Search can be initiated by phenotypes or by genes. A search by phenotype(s) will output genes correlated (≥threshold set in A) to the submitted phenotype(s) and vice versa. (C) Output is displayed as a heatmap. (D) The related hiveplot of each phenotype present in the heatmap is displayed. (E) Filtering options specific for the hiveplots. (F) Tables containing all genes, markers, and metabolites in the hiveplots and their relation. (G) Gene details: known functions, link to other databases, and strongest relations in the BXD dataset with other genes and metabolites. For details, see the online tutorial.
(TIF)

S4 Fig Gene prioritization strategy. (A) Five analysis scores (right; see Fig 3, main text, and Materials and methods) are derived from the actual statistics (left) for (i) *ph*-/*m*QTL FDR-adjusted *p*-value, (ii) *e*QTL q-value, (iii) genetic variant annotation, (iv) Pearson correlation *p*-value, and (v) DE FDR-adjusted *p*-value (from top to bottom). To compute a single gene variant score, we sum the following values for each gene and for each detected variant: splicing = 10; stop-gain = 10; stop-loss = 10; frameshift indel = 10; nonsynonymous = 10 * polyphen2-probability value. (B) We used the central position of the gene to infer the associated *ph*-/*m*QTL analysis score at that position. However, in cases where the associated *cis-e*QTL score or the damaging gene variant score gave a higher value than the *ph-/m*QTL score, the position of the relevant associated marker was used instead. A case of the former is illustrated with the gene *Naa30*. This gene is located near a recombinant region with the central gene position (green arrow) located in a low *ph-/m*QTL associated region, while the *cis-e*QTL-associated marker (red arrow) is located in a highly associated *ph-/m*QTL region. In the case of *Naa30*, its associated *cis-e*QTL score was used. (C) Henikoff weighted scores computed for each phenotype after sleep deprivation. The black line at 0.2 represents the line of equality among the 5 scores (summed weight = 1.0). The *ph-/m*QTL scores generally have higher weights than the other 4 scores because it is the only non-transcript-derived score. The other scores are based in part on the RNA-seq data, and the Henikoff lowers their respective weights because of this dependency. DE, differential expression; *e*QTL, expression quantitative trait locus; FDR, false discovery rate; *ph-/m*QTL, phenotypic/metabolic quantitative trait locus; RNA-seq, RNA sequencing
(TIF)

S5 Fig *Acot11* isoforms. (A) Structure of the 2 *Acot11* isoforms: *NM_001347159* and *NM_025590*. The 2 isoforms differ by a single exon at the start of the transcript. (B) Estimated expression of the 2 *Acot11* isoforms (FPKM) for cortex and liver samples, under the control (“Ctr”) and SD conditions. In cortex, isoform *NM_025590* was highly expressed compared to *NM_001347159*, independent of condition and genotype. Note that for 22 out of the 39 lines, *NM_001347159* expression was near 0. In liver, *NM_025590* was only highly expressed in carriers of the B6 allele for the chromosome 4–associated region for *Acot11* expression, while D2 carriers had close to 0 levels. As in cortex, liver expression of the long isoform was low. Expression was estimated using Cufflinks with option -G for the *Acot11* refseq file. *Acot11*, *acyl-CoA thioesterase 11*; B6, C57BL/6J; D2, DBA/2J; FPKM, fragments per kilobase of transcript per million mapped reads; SD, sleep deprivation
(TIF)

S6 Fig RNA-seq raw gene count. (A) Distribution of raw gene read counts using HTSeq (see Materials and methods) in cortex and liver samples for both the Ctr and SD conditions. Parental strains B6 and D2 are filled with black and brown, respectively. (B) Coefficients of variation in the 4 datasets after normalization. Genes in the liver display a slightly higher coefficient of variation than in cortex. B6, C57BL/6J; D2, DBA/2J; RNA-seq, RNA sequencing; Ctr, control; SD, sleep deprivation
(TIF)

S7 Fig Allelic distribution in the BXD set. (A) Allelic ratios in the 33 BXD lines at all markers. Several genomic regions display a higher genetic imbalance (either toward the D2 or B6 genotype), among which is a region on chromosome 13 containing the QTL *Dps1* (MGI:2135996; see S1 Text). Such imbalance decreases statistical power, making it less likely to map QTLs in these regions. (B) To measure the similarity of the BXD set with C57BL6, we used the Jaccard distance metric with our 11,000 genotypes. We found that a majority of BXD lines have slightly more D2 alleles than B6 alleles. B6, C57BL/6J; D2, DBA/2J; QTL, quantitative trait locus
(TIF)

S1 Table Prioritization scores and weights. Examples of scores and weights obtained with the prioritization algorithm for the 4 phenotypes in the Results section (Examples 1–4) and alpha-aminoadipic acid (top). For each phenotype (and metabolite), the top-5 scored genes are listed per tissue. First column: gene name, columns 2–6: their weighted scores for QTL, *e*QTL, correlation, variation, and DE, column 7: integrated score, column 8: integrated score FDR. Row below fifth gene contains the prioritization weights/phenotype/tissue. Orange highlights the gene that passed the 5% FDR. DE, differential expression; *e*QTL, expression quantitative trait locus; FDR, false discovery rate; QTL, quantitative trait locus.
(XLSX)

S2 Table Top-100 differentially expressed cortical genes after sleep deprivation. Genes are sorted according to fold change. Down-regulated genes are highlighted in gray. Of the 78 genes we considered core molecular components of the sleep homeostatic response in the cortex [34], 13 also made it to this top-100 list (*), and 36 more are among the top 5% most significantly affected genes in the current experiment.
(DOCX)

S3 Table Top-100 differentially expressed liver genes after sleep deprivation. Genes are sorted according to fold change. Down-regulated genes are highlighted in gray.
(DOCX)

S1 Text Lack of reproducibility of the *Dps1* QTL in the old versus the new BXD panel. QTL, quantitative trait locus.
(PDF)

## Abbreviations

α-AAA: alpha-aminoadipic acid
Acot11: *acyl-CoA thioesterase 11*
AMPA: α-amino-3-hydroxy-5-methyl-4-isoxazolepropionic acid
Arc: *activity-regulated cytoskeletal-associated protein*
AMPA-R: α-amino-3-hydroxy-5-methyl-4-isoxazolepropionic acid receptor
ARA: arachidonic acid
ARIL: advanced recombinant inbred line
B6: C57BL/6J mouse
BCAA: branched-chain amino acid
BD-F1: B6D2F1 mouse
CPM: counts per million
Cyp4a32: *Cytochrome P450*, *family 4*, *subfamily a*, *polypeptide 32*
D2: DBA/2J mouse
DB-F1: D2B6F1 mouse
DE: differential expression
DFT: discrete Fourier transform
EEG: electroencephalography
Egr2: *early growth response 2*
EM: Expected-Maximization
EMG: electromyogram
*e*QTL: expression quantitative trait locus
Fam107a: *family with sequence similarity 107*, *A*
FDR: false discovery rate
FFA: free fatty acid
FGF: fibroblast growth factor
FIA: flow injection analysis
GATK: Genome Analysis ToolKit
GRP: genetic reference population
GWAS: Genome-Wide Association Studies
IMPC: International Mouse Phenotyping Consortium
KEGG: Kyoto Encyclopedia of Genes and Genomes
Kif16b: *Kinesin family member 16B*
LC/MS: liquid chromatography/mass spectrometry
LMA: locomotor activity
LOD: logarithm of odds ratio
Mlycd: *malonyl-CoA decarboxylase*
*m*QTL: metabolic quantitative trait locus
NGF: nerve growth factor
NREM: non-REM
PC-aa: phosphatidylcholine acyl-alkyl
*ph*QTL: phenotypic quantitative trait locus
PIR: passive infrared
Pla2g4e: *phospholipase A2*, *group IVE*
Plin4: *Perilipin 4*
QC: quality control
QC2: quality control level 2
QTL: quantitative trait locus
RNA-seq: RNA sequencing
SD: sleep deprivation
SVM: support vector machine
TDW: theta-dominated waking
TPF: theta-peak frequency
Ttll8: *tubulin tyrosine ligase-like family 8*
Wrn: *Werner syndrome RecQ like helicase*
ZT: zeitgeber time

## References

[1] Schmid SM, Hallschmid M, Schultes B. The metabolic burden of sleep loss. The lancet Diabetes & endocrinology. 2015;3(1):52–62. 10.1016/S2213-8587(14)70012-9 24731536

[2] Liu Y, Wheaton AG, Chapman DP, Cunningham TJ, Lu H, Croft JB. Prevalence of Healthy Sleep Duration among Adults—United States, 2014. MMWR Morb Mortal Wkly Rep. 2016;65(6):137–41. Epub 2016/02/20. 10.15585/mmwr.mm6506a1 . 26890214

[3] Xie L, Kang H, Xu Q, Chen MJ, Liao Y, Thiyagarajan M, et al Sleep drives metabolite clearance from the adult brain. Science (New York, NY). 2013;342(6156):373–7. 10.1126/science.1241224 24136970 PMC3880190

[4] Tononi G, Cirelli C. Sleep and the price of plasticity: from synaptic and cellular homeostasis to memory consolidation and integration. Neuron. 2014;81(1):12–34. 10.1016/j.neuron.2013.12.025 24411729 PMC3921176

[5] Maquet P. Sleep function(s) and cerebral metabolism. Behavioural brain research. 1995;69(1–2):75–83. 7546320 10.1016/0166-4328(95)00017-n

[6] Krueger JM, Rector DM, Roy S, Van Dongen HP, Belenky G, Panksepp J. Sleep as a fundamental property of neuronal assemblies. Nature reviews Neuroscience. 2008;9(12):910–9. 10.1038/nrn2521 18985047 PMC2586424

[7] Benington JH, Heller HC. Restoration of brain energy metabolism as the function of sleep. Progress in neurobiology. 1995;45(4):347–60. 7624482 10.1016/0301-0082(94)00057-o

[8] Mang GM, Franken P. Genetic dissection of sleep homeostasis. Current topics in behavioral neurosciences. 2015;25:25–63. 10.1007/7854_2013_270 24338665

[9] Kuna ST, Maislin G, Pack FM, Staley B, Hachadoorian R, Coccaro EF, et al Heritability of performance deficit accumulation during acute sleep deprivation in twins. Sleep. 2012;35(9):1223–33. 10.5665/sleep.2074 22942500 PMC3413799

[10] Franken P, Chollet D, Tafti M. The homeostatic regulation of sleep need is under genetic control. The Journal of neuroscience: the official journal of the Society for Neuroscience. 2001;21(8):2610–21. 10.1523/JNEUROSCI.21-08-02610.2001 PMC6762509 11306614

[11] Lo JC, Groeger JA, Santhi N, Arbon EL, Lazar AS, Hasan S, et al Effects of partial and acute total sleep deprivation on performance across cognitive domains, individuals and circadian phase. PLoS ONE. 2012;7(9):e45987 Epub 2012/10/03. 10.1371/journal.pone.0045987 ; PubMed Central PMCID: PMCPMC3454374. 23029352 PMC3454374

[12] Dissel S, Melnattur K, Shaw PJ. Sleep, Performance, and Memory in Flies. Curr Sleep Med Rep. 2015;1(1):47–54. Epub 2015/06/30. 10.1007/s40675-014-0006-4 ; PubMed Central PMCID: PMCPMC4479072. 26120553 PMC4479072

[13] Urry E, Landolt HP. Adenosine, caffeine, and performance: from cognitive neuroscience of sleep to sleep pharmacogenetics. Curr Top Behav Neurosci. 2015;25:331–66. Epub 2014/02/20. 10.1007/7854_2014_274 . 24549722

[14] Koh K, Joiner WJ, Wu MN, Yue Z, Smith CJ, Sehgal A. Identification of SLEEPLESS, a sleep-promoting factor. Science (New York, NY). 2008;321(5887):372–6. 10.1126/science.1155942 18635795 PMC2771549

[15] Funato H, Miyoshi C, Fujiyama T, Kanda T, Sato M, Wang Z, et al Forward-genetics analysis of sleep in randomly mutagenized mice. Nature. 2016;539(7629):378–83. 10.1038/nature20142 27806374 PMC6076225

[16] Cirelli C, Bushey D, Hill S, Huber R, Kreber R, Ganetzky B, et al Reduced sleep in Drosophila Shaker mutants. Nature. 2005;434(7037):1087–92. 10.1038/nature03486 15858564

[17] Peirce JL, Lu L, Gu J, Silver LM, Williams RW. A new set of BXD recombinant inbred lines from advanced intercross populations in mice. BMC genetics. 2004;5:7 10.1186/1471-2156-5-7 15117419 PMC420238

[18] Civelek M, Lusis AJ. Systems genetics approaches to understand complex traits. Nature reviews Genetics. 2014;15(1):34–48. 10.1038/nrg3575 24296534 PMC3934510

[19] Williams EG, Wu Y, Jha P, Dubuis S, Blattmann P, Argmann CA, et al Systems proteomics of liver mitochondria function. Science (New York, NY). 2016;352(6291). 10.1126/science.aad0189 27284200 PMC10859670

[20] Andreux PAA, Williams EG, Koutnikova H, Houtkooper RH, Champy M-FF, Henry H, et al Systems genetics of metabolism: the use of the BXD murine reference panel for multiscalar integration of traits. Cell. 2012;150(6):1287–99. 10.1016/j.cell.2012.08.012 22939713 PMC3604687

[21] Merkwirth C, Jovaisaite V, Durieux J, Matilainen O, Jordan SD, Quiros PM, et al Two Conserved Histone Demethylases Regulate Mitochondrial Stress-Induced Longevity. Cell. 2016;165(5):1209–23. 10.1016/j.cell.2016.04.012 ; PubMed Central PMCID: PMC4889222. 27133168 PMC4889222

[22] Harbison ST, Carbone MA, Ayroles JF, Stone EA, Lyman RF, Mackay TF. Co-regulated transcriptional networks contribute to natural genetic variation in Drosophila sleep. Nature genetics. 2009;41(3):371–5. 10.1038/ng.330 19234472 PMC2683981

[23] Jiang P, Scarpa JR, Fitzpatrick K, Losic B, Gao VD, Hao K, et al A systems approach identifies networks and genes linking sleep and stress: implications for neuropsychiatric disorders. Cell reports. 2015;11(5):835–48. 10.1016/j.celrep.2015.04.003 25921536 PMC4797932

[24] Baliga NS, Björkegren JL, Boeke JD, Boutros M, Crawford NP, Dudley AMM, et al The State of Systems Genetics in 2017. Cell systems. 2017;4(1):7–15. 10.1016/j.cels.2017.01.005 28125793

[25] Krzywinski M, Birol I, Jones SJ, Marra MA. Hive plots—rational approach to visualizing networks. Briefings in bioinformatics. 2012;13(5):627–44. 10.1093/bib/bbr069 22155641

[26] Hegmann JP, Possidente B. Estimating genetic correlations from inbred strains. Behavior genetics. 1981;11(2):103–14. 7271677 10.1007/BF01065621

[27] Andretic R, Franken P, Tafti M. Genetics of sleep. Annual review of genetics. 2008;42:361–88. 10.1146/annurev.genet.42.110807.091541 18983259

[28] Lander E, Kruglyak L. Genetic dissection of complex traits: guidelines for interpreting and reporting linkage results. Nature genetics. 1995;11(3):241–7. 10.1038/ng1195-241 7581446

[29] Burgess-Herbert SL, Cox A, Tsaih S-WW, Paigen B. Practical applications of the bioinformatics toolbox for narrowing quantitative trait loci. Genetics. 2008;180(4):2227–35. 10.1534/genetics.108.090175 18845850 PMC2600954

[30] Moreau Y, Tranchevent L-CC. Computational tools for prioritizing candidate genes: boosting disease gene discovery. Nature reviews Genetics. 2012;13(8):523–36. 10.1038/nrg3253 22751426

[31] Wu Y, Williams EG, Dubuis S, Mottis A, Jovaisaite V, Houten SM, et al Multilayered genetic and omics dissection of mitochondrial activity in a mouse reference population. Cell. 2014;158(6):1415–30. 10.1016/j.cell.2014.07.039 25215496 PMC4179868

[32] Millstein J, Zhang B, Zhu J, Schadt EE. Disentangling molecular relationships with a causal inference test. BMC Genet. 2009;10:23 Epub 2009/05/29. 10.1186/1471-2156-10-23 ; PubMed Central PMCID: PMCPMC3224661. 19473544 PMC3224661

[33] Pingault JB, O'Reilly PF, Schoeler T, Ploubidis GB, Rijsdijk F, Dudbridge F. Using genetic data to strengthen causal inference in observational research. Nat Rev Genet. 2018 Epub 2018/06/07. 10.1038/s41576-018-0020-3 . 29872216

[34] Mongrain V, Hernandez SA, Pradervand S, Dorsaz S, Curie T, Hagiwara G, et al Separating the contribution of glucocorticoids and wakefulness to the molecular and electrophysiological correlates of sleep homeostasis. Sleep. 2010;33(9):1147–57. 20857860 10.1093/sleep/33.9.1147 PMC2938796

[35] Marini S, Santangeli O, Saarelainen P, Middleton B, Chowdhury N, Skene DJ, et al Abnormalities in the Polysomnographic, Adenosine and Metabolic Response to Sleep Deprivation in an Animal Model of Hyperammonemia. Front Physiol. 2017;8:636 Epub 2017/09/16. 10.3389/fphys.2017.00636 ; PubMed Central PMCID: PMCPMC5583967. 28912724 PMC5583967

[36] Davies SK, Ang JE, Revell VL, Holmes B, Mann A, Robertson FP, et al Effect of sleep deprivation on the human metabolome. Proceedings of the National Academy of Sciences of the United States of America. 2014;111(29):10761–6. 10.1073/pnas.1402663111 25002497 PMC4115565

[37] Giskeødegård GF, Davies SK, Revell VL, Keun H, Skene DJ. Diurnal rhythms in the human urine metabolome during sleep and total sleep deprivation. Scientific reports. 2015;5:14843 10.1038/srep14843 26450397 PMC4598809

[38] Korb E, Finkbeiner S. Arc in synaptic plasticity: from gene to behavior. Trends in neurosciences. 2011;34(11):591–8. 10.1016/j.tins.2011.08.007 21963089 PMC3207967

[39] Wang H, Liu Y, Briesemann M, Yan J. Computational analysis of gene regulation in animal sleep deprivation. Physiological genomics. 2010;42(3):427–36. 10.1152/physiolgenomics.00205.2009 20501693

[40] O'Donovan KJ, Tourtellotte WG, Millbrandt J, Baraban JM. The EGR family of transcription-regulatory factors: progress at the interface of molecular and systems neuroscience. Trends Neurosci. 1999;22(4):167–73. Epub 1999/04/24. . 10203854 10.1016/s0166-2236(98)01343-5

[41] Itabe H, Yamaguchi T, Nimura S, Sasabe N. Perilipins: a diversity of intracellular lipid droplet proteins. Lipids in health and disease. 2017;16(1):83 10.1186/s12944-017-0473-y 28454542 PMC5410086

[42] Rocha C, Papon L, Cacheux W, Marques Sousa P, Lascano V, Tort O, et al Tubulin glycylases are required for primary cilia, control of cell proliferation and tumor development in colon. The EMBO journal. 2014;33(19):2247–60. 10.15252/embj.201488466 25180231 PMC4282510

[43] Schmidt MV, Schülke J-PP, Liebl C, Stiess M, Avrabos C, Bock J, et al Tumor suppressor down-regulated in renal cell carcinoma 1 (DRR1) is a stress-induced actin bundling factor that modulates synaptic efficacy and cognition. Proceedings of the National Academy of Sciences of the United States of America. 2011;108(41):17213–8. 10.1073/pnas.1103318108 21969592 PMC3193257

[44] Masana M, Jukic MM, Kretzschmar A, Wagner KV, Westerholz S, Schmidt MV, et al Deciphering the spatio-temporal expression and stress regulation of Fam107B, the paralog of the resilience-promoting protein DRR1 in the mouse brain. Neuroscience. 2015;290:147–58. 10.1016/j.neuroscience.2015.01.026 25637808

[45] Daan S, Beersma DG, Borbély AA. Timing of human sleep: recovery process gated by a circadian pacemaker. The American journal of physiology. 1984;246(2 Pt 2):83. 10.1152/ajpregu.1984.246.2.R161 6696142

[46] Vassalli A, Franken P. Hypocretin (orexin) is critical in sustaining theta/gamma-rich waking behaviors that drive sleep need. Proceedings of the National Academy of Sciences of the United States of America. 2017;114(27). 10.1073/pnas.1700983114 28630298 PMC5502606

[47] Franken P, Dudley CA, Estill SJ, Barakat M, Thomason R, O'Hara BF, et al NPAS2 as a transcriptional regulator of non-rapid eye movement sleep: genotype and sex interactions. Proceedings of the National Academy of Sciences of the United States of America. 2006;103(18):7118–23. 10.1073/pnas.0602006103 16636276 PMC1459027

[48] Achermann P, Borbely AA. Low-frequency (< 1 Hz) oscillations in the human sleep electroencephalogram. Neuroscience. 1997;81(1):213–22. Epub 1997/09/23. . 9300413 10.1016/s0306-4522(97)00186-3

[49] Vyazovskiy VV, Achermann P, Tobler I. Sleep homeostasis in the rat in the light and dark period. Brain Res Bull. 2007;74(1–3):37–44. Epub 2007/08/09. 10.1016/j.brainresbull.2007.05.001 . 17683787

[50] Landolt HP, Dijk DJ, Gaus SE, Borbely AA. Caffeine reduces low-frequency delta activity in the human sleep EEG. Neuropsychopharmacology. 1995;12(3):229–38. Epub 1995/05/01. 10.1016/0893-133X(94)00079-F . 7612156

[51] Deboer T, Fontana A, Tobler I. Tumor necrosis factor (TNF) ligand and TNF receptor deficiency affects sleep and the sleep EEG. J Neurophysiol. 2002;88(2):839–46. Epub 2002/08/07. 10.1152/jn.2002.88.2.839 . 12163535

[52] Cirelli C, Huber R, Gopalakrishnan A, Southard TL, Tononi G. Locus ceruleus control of slow-wave homeostasis. J Neurosci. 2005;25(18):4503–11. Epub 2005/05/06. 10.1523/JNEUROSCI.4845-04.2005 . 15872097 PMC6725032

[53] Amzica F, Steriade M. Electrophysiological correlates of sleep delta waves. Electroencephalogr Clin Neurophysiol. 1998;107(2):69–83. Epub 1998/09/29. . 9751278 10.1016/s0013-4694(98)00051-0

[54] Dossi RC, Nunez A, Steriade M. Electrophysiology of a slow (0.5–4 Hz) intrinsic oscillation of cat thalamocortical neurones in vivo. J Physiol. 1992;447:215–34. Epub 1992/02/01. ; PubMed Central PMCID: PMCPMC1176033. 1593448 10.1113/jphysiol.1992.sp018999 PMC1176033

[55] Muftuoglu M, Oshima J, von Kobbe C, Cheng W-HH, Leistritz DF, Bohr VA. The clinical characteristics of Werner syndrome: molecular and biochemical diagnosis. Human genetics. 2008;124(4):369–77. 10.1007/s00439-008-0562-0 18810497 PMC4586253

[56] Lee S-YY, Lee H, Kim E-SS, Park S, Lee J, Ahn B. WRN translocation from nucleolus to nucleoplasm is regulated by SIRT1 and required for DNA repair and the development of chemoresistance. Mutation research. 2015;774:40–8. 10.1016/j.mrfmmm.2015.03.001 25801465

[57] Panossian L, Fenik P, Zhu Y, Zhan G, McBurney MW, Veasey S. SIRT1 regulation of wakefulness and senescence-like phenotype in wake neurons. The Journal of neuroscience: the official journal of the Society for Neuroscience. 2011;31(11):4025–36. 10.1523/JNEUROSCI.5166-10.2011 21411645 PMC3065120

[58] Mouchiroud L, Houtkooper RH, Auwerx J. NAD⁺ metabolism: a therapeutic target for age-related metabolic disease. Critical reviews in biochemistry and molecular biology. 2013;48(4):397–408. 10.3109/10409238.2013.789479 23742622 PMC3858599

[59] Li B, Iglesias-Pedraz JM, Chen L-YY, Yin F, Cadenas E, Reddy S, et al Downregulation of the Werner syndrome protein induces a metabolic shift that compromises redox homeostasis and limits proliferation of cancer cells. Aging cell. 2014;13(2):367–78. 10.1111/acel.12181 24757718 PMC3999508

[60] Massip L, Garand C, Turaga RV, Deschênes F, Thorin E, Lebel M. Increased insulin, triglycerides, reactive oxygen species, and cardiac fibrosis in mice with a mutation in the helicase domain of the Werner syndrome gene homologue. Experimental gerontology. 2006;41(2):157–68. 10.1016/j.exger.2005.10.011 16330174

[61] Aumailley L, Garand C, Dubois MJ, Johnson FB, Marette A, Lebel M. Metabolic and Phenotypic Differences between Mice Producing a Werner Syndrome Helicase Mutant Protein and Wrn Null Mice. PLoS ONE. 2015;10(10). 10.1371/journal.pone.0140292 26447695 PMC4598085

[62] Villafuerte G, Miguel-Puga A, Rodríguez EM, Machado S, Manjarrez E, Arias-Carrión O. Sleep deprivation and oxidative stress in animal models: a systematic review. Oxidative medicine and cellular longevity. 2015;2015:234952 10.1155/2015/234952 25945148 PMC4402503

[63] Everson CA, Henchen CJ, Szabo A, Hogg N. Cell injury and repair resulting from sleep loss and sleep recovery in laboratory rats. Sleep. 2014;37(12):1929–40. 10.5665/sleep.4244 25325492 PMC4548518

[64] Dijk D-J. Slow-wave sleep deficiency and enhancement: implications for insomnia and its management. The world journal of biological psychiatry: the official journal of the World Federation of Societies of Biological Psychiatry. 2010;11 Suppl 1:22–8. 10.3109/15622971003637645 20509829

[65] Hasan S, Dauvilliers Y, Mongrain V, Franken P, Tafti M. Age-related changes in sleep in inbred mice are genotype dependent. Neurobiology of aging. 2012;33(1). 10.1016/j.neurobiolaging.2010.05.010 20619936

[66] Maret S, Franken P, Dauvilliers Y, Ghyselinck NB, Chambon P, Tafti M. Retinoic acid signaling affects cortical synchrony during sleep. Science (New York, NY). 2005;310(5745):111–3. 10.1126/science.1117623 16210540

[67] Landolt H-P. Genetic determination of sleep EEG profiles in healthy humans. Progress in brain research. 2011;193:51–61. 10.1016/B978-0-444-53839-0.00004-1 21854955

[68] Franken P. Chapter 4: Genetic mechanisms underlying rhythmic EEG activity during sleep Sleep and Brain Activity, Ed Frank; Oxford: Academic Press; pp 59–89, ISBN: 0123849950. 2012.

[69] Ueno H, Huang X, Tanaka Y, Hirokawa N. KIF16B/Rab14 molecular motor complex is critical for early embryonic development by transporting FGF receptor. Developmental cell. 2011;20(1):60–71. 10.1016/j.devcel.2010.11.008 21238925

[70] Yasuda K, Churchill L, Yasuda T, Blindheim K, Falter M, Krueger JM. Unilateral cortical application of interleukin-1beta (IL1beta) induces asymmetry in fos, IL1beta and nerve growth factor immunoreactivity: implications for sleep regulation. Brain research. 2007;1131(1):44–59. 10.1016/j.brainres.2006.11.051 17184753

[71] Farkhondeh A, Niwa S, Takei Y, Hirokawa N. Characterizing KIF16B in neurons reveals a novel intramolecular "stalk inhibition" mechanism that regulates its capacity to potentiate the selective somatodendritic localization of early endosomes. The Journal of neuroscience: the official journal of the Society for Neuroscience. 2015;35(12):5067–86. 10.1523/JNEUROSCI.4240-14.2015 25810535 PMC6705379

[72] Vyazovskiy VV, Cirelli C, Pfister-Genskow M, Faraguna U, Tononi G. Molecular and electrophysiological evidence for net synaptic potentiation in wake and depression in sleep. Nature neuroscience. 2008;11(2):200–8. 10.1038/nn2035 18204445

[73] Porrino LJ, Daunais JB, Rogers GA, Hampson RE, Deadwyler SA. Facilitation of task performance and removal of the effects of sleep deprivation by an ampakine (CX717) in nonhuman primates. PLoS Biol. 2005;3(9). 10.1371/journal.pbio.0030299 16104830 PMC1188239

[74] Lanté F, Toledo-Salas J-CC, Ondrejcak T, Rowan MJ, Ulrich D. Removal of synaptic Ca^2^+-permeable AMPA receptors during sleep. The Journal of neuroscience: the official journal of the Society for Neuroscience. 2011;31(11):3953–61. 10.1523/JNEUROSCI.3210-10.2011 21411638 PMC6623525

[75] Del Cid-Pellitero E, Plavski A, Mainville L, Jones BE. Homeostatic Changes in GABA and Glutamate Receptors on Excitatory Cortical Neurons during Sleep Deprivation and Recovery. Frontiers in systems neuroscience. 2017;11:17 10.3389/fnsys.2017.00017 28408870 PMC5374161

[76] Boyle J, Stanley N, James LM, Wright N, Johnsen S, Arbon EL, et al Acute sleep deprivation: the effects of the AMPAKINE compound CX717 on human cognitive performance, alertness and recovery sleep. Journal of psychopharmacology. 2012;26(8):1047–57. 10.1177/0269881111405353 . 21940760

[77] Rial Verde EM, Lee-Osbourne J, Worley PF, Malinow R, Cline HT. Increased expression of the immediate-early gene arc/arg3.1 reduces AMPA receptor-mediated synaptic transmission. Neuron. 2006;52(3):461–74. 10.1016/j.neuron.2006.09.031 17088212 PMC3951199

[78] Buzsáki G. Theta oscillations in the hippocampus. Neuron. 2002;33(3):325–40. 11832222 10.1016/s0896-6273(02)00586-x

[79] Boyce R, Glasgow SD, Williams S, Adamantidis A. Causal evidence for the role of REM sleep theta rhythm in contextual memory consolidation. Science (New York, NY). 2016;352(6287):812–6. 10.1126/science.aad5252 27174984

[80] Franken P, Malafosse A, Tafti M. Genetic variation in EEG activity during sleep in inbred mice. The American journal of physiology. 1998;275(4 Pt 2):37. 10.1152/ajpregu.1998.275.4.R1127 9756543

[81] Tafti M, Petit B, Chollet D, Neidhart E, de Bilbao F, Kiss JZ, et al Deficiency in short-chain fatty acid beta-oxidation affects theta oscillations during sleep. Nature genetics. 2003;34(3):320–5. 10.1038/ng1174 12796782

[82] Kroetz DL, Yook P, Costet P, Bianchi P, Pineau T. Peroxisome proliferator-activated receptor alpha controls the hepatic CYP4A induction adaptive response to starvation and diabetes. The Journal of biological chemistry. 1998;273(47):31581–9. . 9813074 10.1074/jbc.273.47.31581

[83] Bazinet RP, Layé S. Polyunsaturated fatty acids and their metabolites in brain function and disease. Nature reviews Neuroscience. 2014;15(12):771–85. 10.1038/nrn3820 25387473

[84] DeCostanzo AJ, Voloshyna I, Rosen ZB, Feinmark SJ, Siegelbaum SA. 12-Lipoxygenase regulates hippocampal long-term potentiation by modulating L-type Ca2+ channels. J Neurosci. 2010;30(5):1822–31. Epub 2010/02/05. 10.1523/JNEUROSCI.2168-09.2010 ; PubMed Central PMCID: PMCPMC2835505. 20130191 PMC2835505

[85] Williams JH, Errington ML, Lynch MA, Bliss TV. Arachidonic acid induces a long-term activity-dependent enhancement of synaptic transmission in the hippocampus. Nature. 1989;341(6244):739–42. Epub 1989/10/26. 10.1038/341739a0 . 2571939

[86] van der Hoeven RS, Steffens JC. Biosynthesis and elongation of short- and medium-chain-length fatty acids. Plant physiology. 2000;122(1):275–82. 10631271 10.1104/pp.122.1.275 PMC58866

[87] Crown SB, Marze N, Antoniewicz MR. Catabolism of Branched Chain Amino Acids Contributes Significantly to Synthesis of Odd-Chain and Even-Chain Fatty Acids in 3T3-L1 Adipocytes. PLoS ONE. 2015;10(12). 10.1371/journal.pone.0145850 26710334 PMC4692509

[88] Newgard CB. Interplay between lipids and branched-chain amino acids in development of insulin resistance. Cell metabolism. 2012;15(5):606–14. 10.1016/j.cmet.2012.01.024 22560213 PMC3695706

[89] Franken P, Malafosse A, Tafti M. Genetic determinants of sleep regulation in inbred mice. Sleep. 1999;22(2):155–69. 10201060

[90] Cohen DE. New players on the metabolic stage: How do you like Them Acots? Adipocyte. 2013;2(1):3–6. 10.4161/adip.21853 23700546 PMC3661129

[91] Zhang Y, Li Y, Niepel MW, Kawano Y, Han S, Liu S, et al Targeted deletion of thioesterase superfamily member 1 promotes energy expenditure and protects against obesity and insulin resistance. Proceedings of the National Academy of Sciences of the United States of America. 2012;109(14):5417–22. 10.1073/pnas.1116011109 22427358 PMC3325675

[92] Broussard JL, Chapotot F, Abraham V, Day A, Delebecque F, Whitmore HR, et al Sleep restriction increases free fatty acids in healthy men. Diabetologia. 2015;58(4):791–8. 10.1007/s00125-015-3500-4 25702040 PMC4358810

[93] Boden G. Effects of free fatty acids (FFA) on glucose metabolism: significance for insulin resistance and type 2 diabetes. Experimental and clinical endocrinology & diabetes: official journal, German Society of Endocrinology [and] German Diabetes Association. 2003;111(3):121–4. 10.1055/s-2003-39781 12784183

[94] DeFronzo RA. Dysfunctional fat cells, lipotoxicity and type 2 diabetes. International journal of clinical practice Supplement. 2004;(143):9–21. 10.1111/j.1368-504x.2004.00389.x 16035392

[95] Spiegel K, Knutson K, Leproult R, Tasali E, Van Cauter E. Sleep loss: a novel risk factor for insulin resistance and Type 2 diabetes. Journal of applied physiology (Bethesda, Md: 1985). 2005;99(5):2008–19. 10.1152/japplphysiol.00660.2005 16227462

[96] Buxton OM, Pavlova M, Reid EW, Wang W, Simonson DC, Adler GK. Sleep restriction for 1 week reduces insulin sensitivity in healthy men. Diabetes. 2010;59(9):2126–33. 10.2337/db09-0699 20585000 PMC2927933

[97] Wang X, Pandey AK, Mulligan MK, Williams EG, Mozhui K, Li Z, et al Joint mouse-human phenome-wide association to test gene function and disease risk. Nature communications. 2016;7:10464 10.1038/ncomms10464 ; PubMed Central PMCID: PMC4740880. 26833085 PMC4740880

[98] Arnar DO, Andersen K, Thorgeirsson G. Genetics of cardiovascular diseases: lessons learned from a decade of genomics research in Iceland. Scandinavian cardiovascular journal: SCJ. 2016;50(5–6):260–5. 10.1080/14017431.2016.1230679 . 27572422

[99] Milani L, Leitsalu L, Metspalu A. An epidemiological perspective of personalized medicine: the Estonian experience. Journal of internal medicine. 2015;277(2):188–200. 10.1111/joim.12320 ; PubMed Central PMCID: PMC4329410. 25339628 PMC4329410

[100] Peltonen L, Palotie A, Lange K. Use of population isolates for mapping complex traits. Nat Rev Genet. 2000;1(3):182–90. 10.1038/35042049 . 11252747

[101] Franken P, Tafti M. Genetics of sleep and sleep disorders. Frontiers in bioscience: a journal and virtual library. 2003;8:97. 10.2741/1084 12700094

[102] He D, Parida L. Muse: A Multi-Locus Sampling-Based Epistasis Algorithm for Quantitative Genetic Trait Prediction. Pacific Symposium on Biocomputing Pacific Symposium on Biocomputing. 2016;22:426–37. 10.1142/9789813207813_0040 . 27896995

[103] Llinares-Lopez F, Grimm DG, Bodenham DA, Gieraths U, Sugiyama M, Rowan B, et al Genome-wide detection of intervals of genetic heterogeneity associated with complex traits. Bioinformatics. 2015;31(12):i240–9. Epub 2015/06/15. 10.1093/bioinformatics/btv263 ; PubMed Central PMCID: PMCPMC4559912. 26072488 PMC4559912

[104] Wilkinson MD, Dumontier M, Aalbersberg IJ, Appleton G, Axton M, Baak A, et al The FAIR Guiding Principles for scientific data management and stewardship. Scientific data. 2016;3:160018 10.1038/sdata.2016.18 ; PubMed Central PMCID: PMC4792175. 26978244 PMC4792175

[105] Mang GM, Franken P. Sleep and EEG Phenotyping in Mice. Current protocols in mouse biology. 2012;2(1):55–74. 10.1002/9780470942390.mo110126 26069005

[106] Meyer D, Dimitriadou E, Hornik K, Weingessel A, Leisch F. e1071: Misc Functions of the Department of Statistics (e1071), TU Wien. http://CRANR-projectorg/package=e1071. 2014.

[107] Kuhn M, Wing J, Weston S, Williams A, Keefer C, Engelhardt A, et al. caret: Classification and Regression Training. http://CRANR-projectorg/package=caret. 2014.

[108] Welsh DK, Richardson GS, Dement WC. A circadian rhythm of hippocampal theta activity in the mouse. Physiology & behavior. 1985;35(4):533–8. 4070426 10.1016/0031-9384(85)90136-2

[109] Ryan LJ. Characterization of cortical spindles in DBA/2 and C57BL/6 inbred mice. Brain research bulletin. 1984;13(4):549–58. 6441615 10.1016/0361-9230(84)90037-6

[110] Isherwood CM, Van der Veen DR, Johnston JD, Skene DJ. Twenty-four-hour rhythmicity of circulating metabolites: effect of body mass and type 2 diabetes. FASEB J. 2017 Epub 2017/08/20. 10.1096/fj.201700323R . 28821636 PMC5690388

[111] Picard A, Soyer J, Berney X, Tarussio D, Quenneville S, Jan M, et al A Genetic Screen Identifies Hypothalamic Fgf15 as a Regulator of Glucagon Secretion. Cell reports. 2016;17(7):1795–806. 10.1016/j.celrep.2016.10.041 27829151 PMC5120348

[112] Dobin A, Davis CA, Schlesinger F, Drenkow J, Zaleski C, Jha S, et al STAR: ultrafast universal RNA-seq aligner. Bioinformatics. 2013;29(1):15–21. Epub 2012/10/30. 10.1093/bioinformatics/bts635 ; PubMed Central PMCID: PMCPMC3530905. 23104886 PMC3530905

[113] Anders S, Pyl PT, Huber W. HTSeq—a Python framework to work with high-throughput sequencing data. Bioinformatics. 2015;31(2):166–9. 10.1093/bioinformatics/btu638 ; PubMed Central PMCID: PMCPMC4287950. 25260700 PMC4287950

[114] Robinson M, Oshlack A. A scaling normalization method for differential expression analysis of RNA-seq data. 2010. 10.1186/gb-2010-11-3-r25 PMC2864565 20196867

[115] Ritchie ME, Phipson B, Wu D, Hu Y, Law CW, Shi W, et al limma powers differential expression analyses for RNA-sequencing and microarray studies. Nucleic Acids Res. 2015;43(7):e47 10.1093/nar/gkv007 ; PubMed Central PMCID: PMCPMC4402510. 25605792 PMC4402510

[116] Law CW, Chen JC, Shi W, Smyth GK. voom: precision weights unlock linear model analysis tools for RNA-seq read counts. 2014. 10.1186/gb-2014-15-2-r29 PMC4053721 24485249

[117] McKenna A, Hanna M, Banks E, Sivachenko A, Cibulskis K, Kernytsky A, et al The Genome Analysis Toolkit: a MapReduce framework for analyzing next-generation DNA sequencing data. Genome Res. 2010;20(9):1297–303. 10.1101/gr.107524.110 ; PubMed Central PMCID: PMCPMC2928508. 20644199 PMC2928508

[118] DePristo MA, Banks E, Poplin R, Garimella KV, Maguire JR, Hartl C, et al A framework for variation discovery and genotyping using next-generation DNA sequencing data. Nat Genet. 2011;43(5):491–8. 10.1038/ng.806 ; PubMed Central PMCID: PMCPMC3083463. 21478889 PMC3083463

[119] Van der Auwera GA, Carneiro MO, Hartl C, Poplin R, Del Angel G, Levy-Moonshine A, et al From FastQ data to high confidence variant calls: the Genome Analysis Toolkit best practices pipeline. Curr Protoc Bioinformatics. 2013;43:11 0 1–33. 10.1002/0471250953.bi1110s43 ; PubMed Central PMCID: PMCPMC4243306. 25431634 PMC4243306

[120] Broman KW, Wu H, Sen S, Churchill GA. R/qtl: QTL mapping in experimental crosses. Bioinformatics. 2003;19(7):889–90. 10.1093/bioinformatics/btg112 12724300

[121] Broman KW, Sen S. A Guide to QTL Mapping with R/qtl: New York: Springer; 2009.

[122] Ongen H, Buil A, Brown AA, Dermitzakis ET, Delaneau O. Fast and efficient QTL mapper for thousands of molecular phenotypes. Bioinformatics. 2016;32(10):1479–85. Epub 2015/12/29. 10.1093/bioinformatics/btv722 ; PubMed Central PMCID: PMCPMC4866519. 26708335 PMC4866519

[123] Storey JD, Bass AJ, Dabney A, Robinson D. qvalue: Q-value estimation for false discovery rate control. R package version 2.8.0, 2015.

[124] Schupbach T, Xenarios I, Bergmann S, Kapur K. FastEpistasis: a high performance computing solution for quantitative trait epistasis. Bioinformatics. 2010;26(11):1468–9. 10.1093/bioinformatics/btq147 20375113 PMC2872003

[125] Wang K, Li M, Hakonarson H. ANNOVAR: functional annotation of genetic variants from high-throughput sequencing data. Nucleic Acids Res. 2010;38(16):e164 10.1093/nar/gkq603 ; PubMed Central PMCID: PMCPMC2938201. 20601685 PMC2938201

[126] Adzhubei IA, Schmidt S, Peshkin L, Ramensky VE, Gerasimova A, Bork P, et al A method and server for predicting damaging missense mutations. Nat Methods. 2010;7(4):248–9. 10.1038/nmeth0410-248 ; PubMed Central PMCID: PMCPMC2855889. 20354512 PMC2855889

