# Supplementary figures and images for: A systems genetics resource and analysis of sleep regulation in the mouse
Source: PLoS Biol. 2018 Aug 9;16(8):e2005750. doi: 10.1371/journal.pbio.2005750 (PMC6085075; doi:10.1371/journal.pbio.2005750)

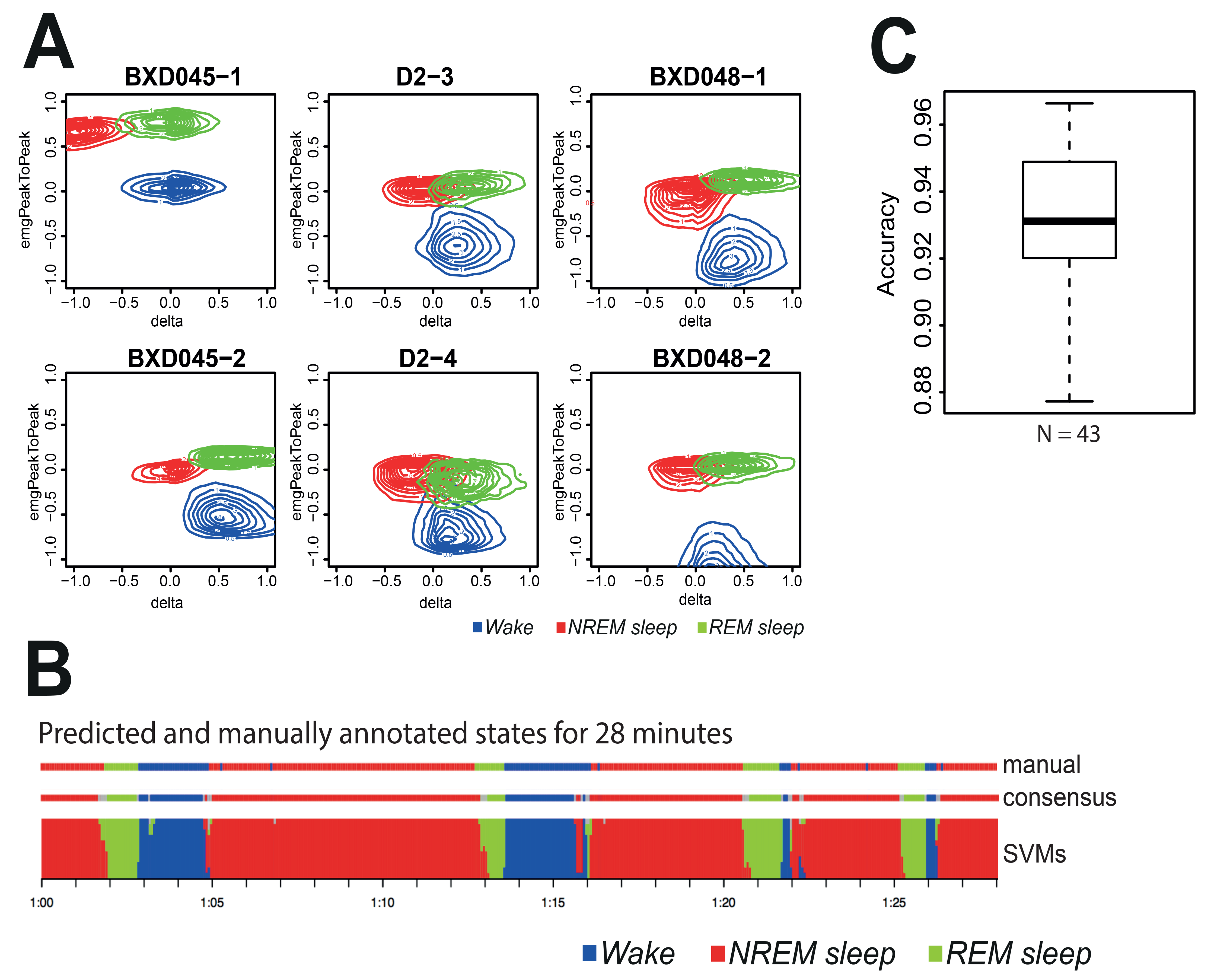

Supplement: S1 Fig — (A) Comparison of the normalized signal for 2 individual mice (top and bottom rows) of 2 BXD lines (left and right) and 1 parental line (DBA/2J; Middle), visually annotated by an expert scorer. Plotted are the peak-to-peak EMG amplitude (y-axis) against EEG delta (1.0–4.0 Hz) power (x-axis). (B) Example of predicted sleep-wake states of a representative 28 min section (420 four-s epochs) of mouse BXD045-1. Top row: state manually assigned by the expert. Second row: consensus of the automated prediction. Third row: results obtained for 11 distinct SVM predictors from which the consensus prediction is derived. (C) Accuracy values of the prediction for the 43 mice for which the 4 d recordings were fully annotated by the expert. The SVMs were trained on the R1 recording and then used to predict sleep-wake state for days B1, B2, and R2. Predicted sleep-wake states were compared to manual annotation using a confusion matrix (see Materials and methods). EEG, electroencephalography; EMG, electromyography; SVM, support vector machine (TIF) [file pbio.2005750.s001.tif]

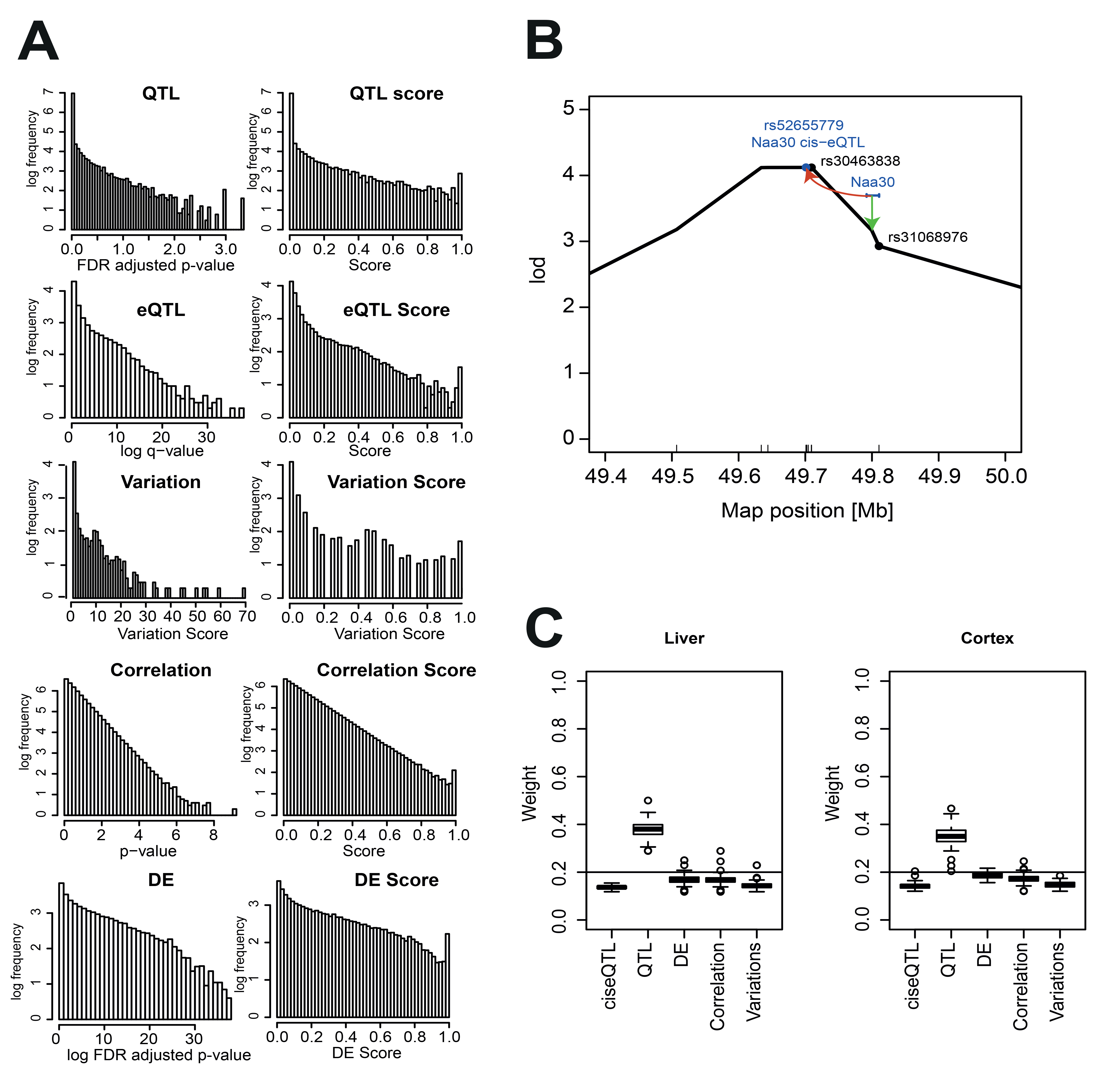

Supplement: S4 Fig — (A) Five analysis scores (right; see Fig 3, main text, and Materials and methods) are derived from the actual statistics (left) for (i) ph-/mQTL FDR-adjusted p-value, (ii) eQTL q-value, (iii) genetic variant annotation, (iv) Pearson correlation p-value, and (v) DE FDR-adjusted p-value (from top to bottom). To compute a single gene variant score, we sum the following values for each gene and for each detected variant: splicing = 10; stop-gain = 10; stop-loss = 10; frameshift indel = 10; nonsynonymous = 10 * polyphen2-probability value. (B) We used the central position of the gene to infer the associated ph-/mQTL analysis score at that position. However, in cases where the associated cis-eQTL score or the damaging gene variant score gave a higher value than the ph-/mQTL score, the position of the relevant associated marker was used instead. A case of the former is illustrated with the gene Naa30. This gene is located near a recombinant region with the central gene position (green arrow) located in a low ph-/mQTL associated region, while the cis-eQTL-associated marker (red arrow) is located in a highly associated ph-/mQTL region. In the case of Naa30, its associated cis-eQTL score was used. (C) Henikoff weighted scores computed for each phenotype after sleep deprivation. The black line at 0.2 represents the line of equality among the 5 scores (summed weight = 1.0). The ph-/mQTL scores generally have higher weights than the other 4 scores because it is the only non-transcript-derived score. The other scores are based in part on the RNA-seq data, and the Henikoff lowers their respective weights because of this dependency. DE, differential expression; eQTL, expression quantitative trait locus; FDR, false discovery rate; ph-/mQTL, phenotypic/metabolic quantitative trait locus; RNA-seq, RNA sequencing (TIF) [file pbio.2005750.s004.tif]

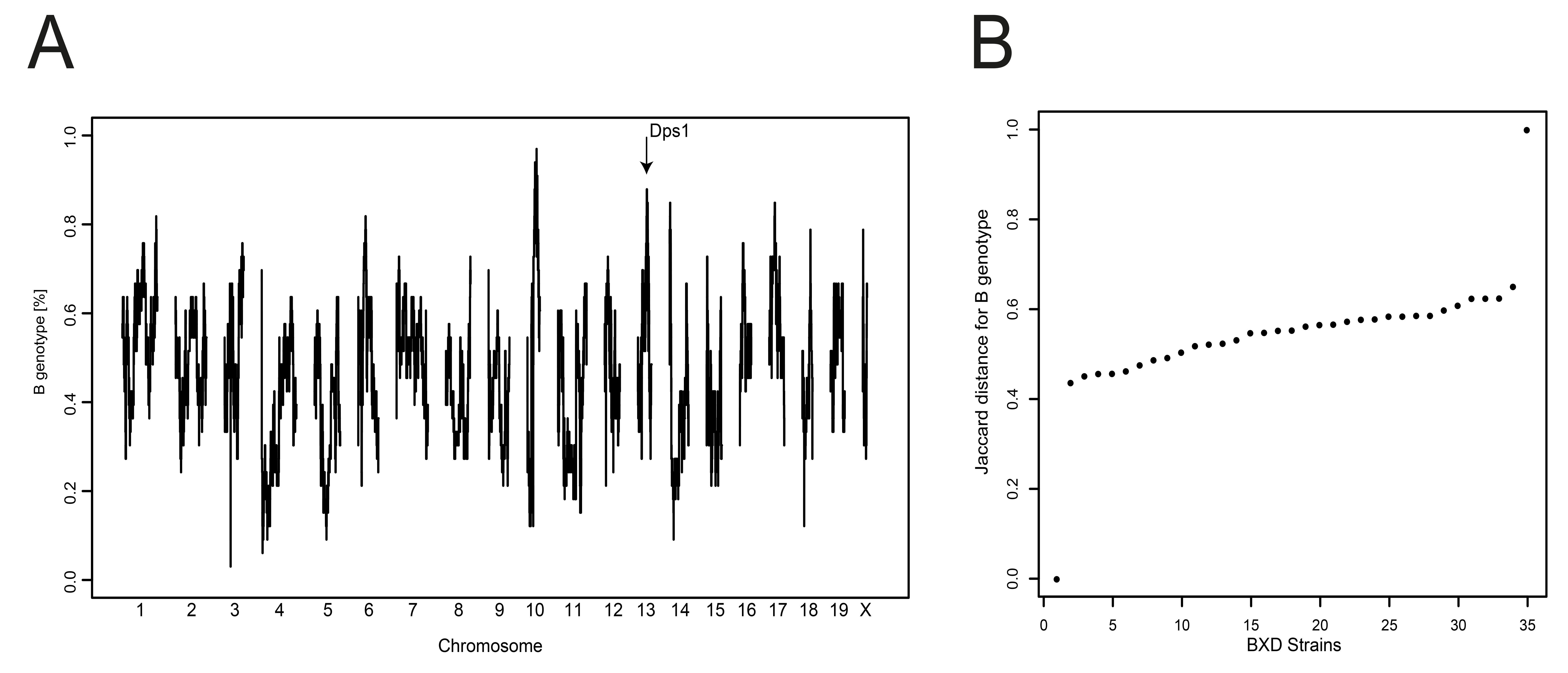

Supplement: S7 Fig — (A) Allelic ratios in the 33 BXD lines at all markers. Several genomic regions display a higher genetic imbalance (either toward the D2 or B6 genotype), among which is a region on chromosome 13 containing the QTL Dps1 (MGI:2135996; see S1 Text). Such imbalance decreases statistical power, making it less likely to map QTLs in these regions. (B) To measure the similarity of the BXD set with C57BL6, we used the Jaccard distance metric with our 11,000 genotypes. We found that a majority of BXD lines have slightly more D2 alleles than B6 alleles. B6, C57BL/6J; D2, DBA/2J; QTL, quantitative trait locus (TIF) [file pbio.2005750.s007.tif]
