# Supplementary material for: A systems genetics resource and analysis of sleep regulation in the mouse
Source: PLoS Biol. 2018 Aug 9;16(8):e2005750. doi: 10.1371/journal.pbio.2005750 (PMC6085075; doi:10.1371/journal.pbio.2005750)
Supplement: S2 Table — Genes are sorted according to fold change. Down-regulated genes are highlighted in gray. Of the 78 genes we considered core molecular components of the sleep homeostatic response in the cortex [34], 13 also made it to this top-100 list (*), and 36 more are among the top 5% most significantly affected genes in the current experiment. (DOCX) [file pbio.2005750.s009.docx]

| **Gene** | **Log2 FC** | **Adjusted p-value** |  | **Gene** | **Log2 FC** | **Adjusted p-value** |  | **Gene** | **Log2 FC** | **Adjusted p-value** |  | **Gene** | **Log2 FC** | **Adjusted p-value** |
| --- | --- | --- | --- | --- | --- | --- | --- | --- | --- | --- | --- | --- | --- | --- |
| *Plin4* | 2.95 | 4.66E-24 |  | *Nr4a1** | 1.52 | 4.78E-28 |  | *Zglp1* | 1.36 | 3.09E-24 |  | *8430408G22Rik* | 1.22 | 1.76E-14 |
| *Arc** | 2.90 | 1.64E-32 |  | *Serinc2* | 1.49 | 2.10E-27 |  | *Slc23a3* | 1.35 | 6.24E-21 |  | *Hist2h3c1* | 1.22 | 1.66E-25 |
| *Egr2* | 2.88 | 3.74E-25 |  | *Dusp4* | 1.48 | 4.29E-29 |  | *Stac3* | 1.35 | 6.56E-29 |  | *Derl3* | 1.22 | 1.02E-20 |
| *Tekt4* | 2.23 | 2.14E-16 |  | *Tmem252* | 1.48 | 1.72E-16 |  | *Cstad* | -1.35 | 2.47E-24 |  | *BC021891* | -1.22 | 6.32E-30 |
| *Fos** | 2.20 | 3.56E-24 |  | *Sik1* | 1.47 | 1.29E-27 |  | *Hspa1b** | 1.34 | 1.72E-25 |  | *A730020M07Rik* | 1.21 | 6.27E-16 |
| *Plekhg4* | 2.12 | 7.81E-34 |  | *Npas4** | 1.47 | 2.86E-26 |  | *Htr5b* | 1.33 | 4.46E-19 |  | *Junb* | 1.20 | 6.88E-25 |
| *Slco1b2* | 2.08 | 3.40E-24 |  | *Samd3* | -1.46 | 3.29E-22 |  | *Hspa5** | 1.32 | 6.58E-38 |  | *Arhgef33* | -1.20 | 1.36E-07 |
| *Gh* | -1.98 | 4.21E-05 |  | *Trib1* | 1.45 | 4.99E-34 |  | *Fam150b* | 1.31 | 2.20E-22 |  | *Pcsk9* | -1.19 | 1.64E-24 |
| *Tnfrsf25* | 1.96 | 3.39E-34 |  | *Cbln4** | 1.45 | 1.06E-34 |  | *Cyr61* | 1.29 | 1.09E-21 |  | *Mir6982* | 1.19 | 8.40E-17 |
| *Misp* | 1.90 | 7.87E-19 |  | *0610040B10Rik* | -1.45 | 1.11E-20 |  | *Map3k6* | 1.28 | 1.65E-21 |  | *Npbwr1* | -1.19 | 1.30E-19 |
| *Rtp1* | 1.87 | 2.27E-15 |  | *1700001L05Rik* | -1.45 | 4.28E-33 |  | *Dusp5* | 1.28 | 7.41E-27 |  | *Pla2g4e* | -1.19 | 1.52E-18 |
| *1700102P08Rik* | 1.80 | 9.32E-29 |  | *Hspb1* | 1.44 | 2.34E-20 |  | *Gck* | -1.28 | 6.61E-18 |  | *Nr4a3** | 1.19 | 2.42E-22 |
| *Fosb* | 1.75 | 7.68E-29 |  | *Fosl2* | 1.44 | 9.67E-29 |  | *Itga10* | 1.27 | 3.75E-34 |  | *Insl5* | 1.18 | 2.14E-17 |
| *Maff* | 1.72 | 2.22E-16 |  | *Mybpc3* | -1.43 | 1.04E-11 |  | *Alox12* | -1.27 | 9.03E-15 |  | *Vwa3a* | -1.17 | 8.23E-32 |
| *Tm6sf2* | 1.68 | 3.63E-27 |  | *Rasl11a* | 1.43 | 2.85E-28 |  | *Cirbp** | -1.27 | 6.52E-34 |  | *AW549542* | -1.17 | 5.98E-22 |
| *Sdf2l1** | 1.66 | 5.32E-37 |  | *Dok3* | 1.43 | 6.29E-34 |  | *Ptgs2* | 1.27 | 4.21E-24 |  | *Map3k19* | 1.17 | 2.95E-27 |
| *Bace2* | -1.63 | 3.10E-28 |  | *Gkn3* | -1.42 | 3.71E-20 |  | *Mdga1* | 1.26 | 3.87E-31 |  | *Spry4* | 1.17 | 2.47E-32 |
| *Cdkn1a* | 1.62 | 1.29E-24 |  | *Parpbp* | -1.42 | 3.56E-20 |  | *Cd28* | -1.26 | 8.20E-11 |  | *Espnl* | -1.17 | 2.36E-15 |
| *Klhdc9* | -1.60 | 3.33E-24 |  | *Dio2** | 1.40 | 1.47E-33 |  | *Neurog2* | -1.25 | 3.64E-19 |  | *Tmem82* | 1.16 | 1.22E-11 |
| *Noxred1* | 1.59 | 5.36E-28 |  | *Egr1** | 1.40 | 5.36E-28 |  | *Hist1h2be* | 1.25 | 6.13E-27 |  | *Gli1* | -1.16 | 1.55E-22 |
| *Rasd1* | 1.59 | 4.83E-27 |  | *Cml5* | 1.39 | 2.16E-22 |  | *Fat2* | -1.25 | 1.72E-02 |  | *Tmod4* | 1.16 | 2.35E-15 |
| *Lrrc29* | -1.55 | 4.07E-25 |  | *Gpr3* | 1.37 | 9.92E-29 |  | *Pglyrp1* | 1.25 | 3.95E-29 |  | *Hfe2* | -1.15 | 1.65E-22 |
| *Fam83d* | 1.55 | 5.22E-22 |  | *Acr* | -1.37 | 3.20E-26 |  | *Gm16062* | 1.24 | 2.79E-21 |  | *4930426L09Rik* | -1.15 | 9.27E-16 |
| *Egr3** | 1.53 | 2.59E-34 |  | *Gm1141* | 1.37 | 8.80E-17 |  | *Hif3a* | 1.24 | 2.56E-23 |  | *Nlrc4* | -1.15 | 1.02E-13 |
| *Timp1* | 1.52 | 1.14E-08 |  | *B430319G15Rik* | -1.37 | 8.64E-19 |  | *Arl4d* | 1.22 | 7.45E-32 |  | *Rho* | 1.14 | 6.10E-14 |
