# Supplementary material for: A systems genetics resource and analysis of sleep regulation in the mouse
Source: PLoS Biol. 2018 Aug 9;16(8):e2005750. doi: 10.1371/journal.pbio.2005750 (PMC6085075; doi:10.1371/journal.pbio.2005750)
Supplement: S3 Table — Genes are sorted according to fold change. Down-regulated genes are highlighted in gray. (DOCX) [file pbio.2005750.s010.docx]

| **Gene** | **Log2 FC** | **Adjusted p-value** |  | **Gene** | **Log2 FC** | **Adjusted p-value** |  | **Gene** | **Log2 FC** | **Adjusted p-value** |  | **Gene** | **Log2 FC** | **Adjusted p-value** |
| --- | --- | --- | --- | --- | --- | --- | --- | --- | --- | --- | --- | --- | --- | --- |
| *Ttll8* | 2.94 | 2.82E-21 |  | *Fmo3* | 1.59 | 1.24E-09 |  | *Hsph1* | 1.33 | 1.20E-32 |  | *Lefty1* | -1.15 | 7.73E-13 |
| *Fam107a* | 2.62 | 2.07E-13 |  | *Pnpla5* | -1.58 | 2.29E-06 |  | *Tchh* | 1.32 | 6.72E-12 |  | *Smpd3* | -1.14 | 1.13E-11 |
| *Fam65b* | -2.49 | 2.95E-22 |  | *Slc5a3* | 1.54 | 1.20E-30 |  | *Fbxl22* | 1.28 | 1.73E-13 |  | *2810403D21Rik* | -1.14 | 5.64E-13 |
| *Lmod2* | 2.35 | 7.65E-14 |  | *Hist2h3c2* | 1.54 | 9.78E-20 |  | *Syt11* | 1.27 | 4.28E-26 |  | *P4ha2* | 1.14 | 6.88E-26 |
| *Hist1h1e* | 2.34 | 3.56E-22 |  | *Cirbp* | -1.53 | 8.26E-32 |  | *Esrrg* | -1.26 | 1.86E-15 |  | *Cand2* | -1.12 | 2.97E-10 |
| *Zbtb16* | 2.22 | 1.24E-21 |  | *1810046K07Rik* | -1.53 | 2.77E-25 |  | *Cyp2b10* | 1.25 | 5.87E-07 |  | *Syn3* | -1.12 | 9.65E-20 |
| *Herc3* | -2.01 | 4.90E-29 |  | *Lrtm1* | -1.52 | 3.48E-21 |  | *Mt2* | 1.22 | 2.94E-08 |  | *Unc13a* | -1.12 | 2.40E-10 |
| *Nfe2* | -1.96 | 4.64E-14 |  | *Rps6kl1* | -1.49 | 2.82E-10 |  | *Pde4d* | -1.21 | 1.55E-17 |  | *AA986860* | 1.11 | 4.68E-13 |
| *Slc51b* | -1.95 | 7.85E-13 |  | *Tnfaip8l3* | -1.49 | 3.28E-15 |  | *Plin4* | 1.21 | 2.44E-11 |  | *Nlrp12* | 1.11 | 5.10E-20 |
| *1700092C10Rik* | -1.92 | 6.44E-20 |  | *Themis* | -1.46 | 5.95E-13 |  | *Fam171b* | -1.21 | 7.84E-08 |  | *1700030J22Rik* | 1.11 | 2.26E-11 |
| *Mroh6* | 1.81 | 7.37E-14 |  | *Hist2h3c1* | 1.46 | 1.71E-27 |  | *Chrna4* | -1.20 | 4.67E-07 |  | *Cd79b* | -1.11 | 4.73E-14 |
| *Rad51c* | 1.81 | 7.22E-22 |  | *Pfkfb3* | 1.45 | 5.04E-25 |  | *Tmc7* | 1.20 | 1.19E-14 |  | *Dnah5* | -1.10 | 5.53E-08 |
| *Dmbt1* | -1.77 | 4.85E-02 |  | *Gm16063* | -1.44 | 5.51E-16 |  | *4931408D14Rik* | -1.20 | 4.93E-22 |  | *Nr4a2* | 1.10 | 6.65E-12 |
| *5930430L01Rik* | -1.73 | 1.06E-21 |  | *Map3k6* | 1.44 | 3.54E-16 |  | *1700040L02Rik* | -1.19 | 2.11E-20 |  | *Fos* | 1.10 | 6.83E-12 |
| *D130043K22Rik* | -1.72 | 1.88E-21 |  | *Lrrc16a* | -1.43 | 1.02E-28 |  | *Snhg11* | -1.18 | 1.51E-14 |  | *C330021F23Rik* | -1.09 | 1.46E-18 |
| *Hspa1a* | 1.70 | 7.72E-12 |  | *Ell3* | -1.40 | 1.46E-19 |  | *Inhbb* | 1.18 | 4.01E-16 |  | *Gnat1* | -1.09 | 7.00E-22 |
| *Fam83f* | -1.70 | 2.05E-19 |  | *Ctgf* | 1.39 | 1.31E-16 |  | *Dio3os* | 1.18 | 7.46E-13 |  | *Pde6c* | -1.08 | 8.21E-09 |
| *Hspa1b* | 1.68 | 9.77E-20 |  | *4732491K20Rik* | -1.37 | 4.91E-14 |  | *Prss53* | 1.17 | 6.03E-23 |  | *Cdkn1a* | 1.08 | 2.91E-07 |
| *Chd3os* | 1.66 | 1.21E-22 |  | *1810053B23Rik* | 1.36 | 3.82E-08 |  | *Esco2* | 1.16 | 4.27E-15 |  | *B930025P03Rik* | 1.08 | 3.27E-11 |
| *E2f8* | 1.65 | 4.35E-19 |  | *Ms4a1* | -1.36 | 4.55E-08 |  | *Mfsd2a* | 1.16 | 1.45E-17 |  | *Dlgap1* | -1.08 | 2.13E-16 |
| *Pnpla3* | -1.65 | 4.32E-08 |  | *Scara5* | 1.34 | 2.38E-20 |  | *Fam184b* | -1.16 | 1.09E-10 |  | *Eif4ebp3* | 1.08 | 9.96E-25 |
| *Derl3* | 1.64 | 5.23E-16 |  | *Zfp618* | -1.34 | 6.12E-14 |  | *Dnah17* | -1.15 | 8.47E-10 |  | *Adamts7* | -1.07 | 9.37E-21 |
| *Cd79a* | -1.60 | 3.84E-11 |  | *Ppp1r3g* | 1.34 | 2.48E-08 |  | *Slc1a4* | -1.15 | 2.18E-18 |  | *Gramd1c* | -1.07 | 8.88E-26 |
| *Tes* | 1.59 | 8.16E-21 |  | *Fkbp5* | 1.34 | 6.55E-21 |  | *Gng7* | -1.15 | 5.33E-14 |  | *Tbc1d8* | 1.06 | 1.20E-23 |
| *Nptx1* | -1.59 | 1.29E-12 |  | *Hspb1* | 1.34 | 2.06E-23 |  | *1700056E22Rik* | -1.15 | 2.29E-14 |  | *Fam46c* | 1.06 | 2.02E-18 |
