# Supplementary material for: A systems genetics resource and analysis of sleep regulation in the mouse
Source: PLoS Biol. 2018 Aug 9;16(8):e2005750. doi: 10.1371/journal.pbio.2005750 (PMC6085075; doi:10.1371/journal.pbio.2005750)
Supplement: S1 Text — QTL, quantitative trait locus. (PDF) [file pbio.2005750.s011.pdf]

In the older BXD panel (BXD1-42/*TyJ*), we previously mapped a significant QTL on chromosome 13 (*Dsp1*; MGI: 2135996) that could explain a large portion of the variance in the increase (or gain) in EEG delta power (compared to the lowest values reached in baseline between ZT8-12) during NREM sleep immediately following a sleep deprivation (Franken *et al*., 2001). Subsequent studies identified *Homer1* as a credible candidate gene (Mackiewicz *et al.*, 2008; Maret *et al.*, 2007), and specifically its short isoform *Homer1a* might mechanistically link sleep homeostatic drive to one proposed function of NREM sleep, namely synaptic down-scaling (Diering *et al.*, 2017). In the current BXD set (BXD43-161/*RwwJ*), we could not replicate the *Dps1* QTL. Among the many possible explanations for the differences between the two studies are (i) the strong bias in allele frequency toward the B6 genotype at the *Dps1* region in the 33 BXD lines we used in the current study (28/33 lines, S11 Fig), a situation which decreases statistical power for QTL detection and is also thought to contribute to replication issues among QTL studies (Gatti *et al*., 2009), (ii) the two BXD sets were derived from two different DBA/2 sub-strains [i.e., DBA/2Rj *vs.* DBA/2J; new *vs.* old (Shin *et al.*, 2014)], (iii) genetic drift (Reardon, 2017; Shifman *et al.*, 2006), (iv) interaction with other loci that are differently represented in the two BXD sets, and (v) differences in phenotyping. In an attempt to address some of these issues, we re-phenotyped two of the older BXD lines, namely BXD5/*TyJ* and BXD32/*TyJ*, which 17 years earlier gave the highest and lowest EEG delta power increase after sleep deprivation, respectively (Franken *et al*., 2001). While for BXD5, which carries a B6-allele at *Dps1* as well as for the B6 parental strain, we obtained a close to perfect match (B6: 221.2 vs. 223.1%, BXD5: 223.3 vs. 223.6% over baseline, current *vs.* previous), for BXD32, a D2-allele carrier at the *Dsp1* locus, and the D2 parental line we observed notable discrepancies (D2: 168.9 *vs.* 179.5%; BXD32: 159.2 *vs.* 133.0%, current *vs.* previous). Thus each BXD set must be regarded as a GRP (Genetic Reference Population) in its own right and QTL analyses can lead to different sets of equally valid genetic associations due to genetic drift and other above-mentioned reasons.

Diering GH, Nirujogi RS, Roth RH, Worley PF, Pandey A, Huganir RL (2017) Homer1a drives homeostatic scaling- down of excitatory synapses during sleep. Science (New York, NY) 355: 511-515

Franken P, Chollet D, Tafti M (2001) The homeostatic regulation of sleep need is under genetic control. The Journal of neuroscience : the official journal of the Society for Neuroscience 21: 2610-2621

Gatti DM, Harrill AH, Wright FA, Threadgill DW, Rusyn I (2009) Replication and narrowing of gene expression quantitative trait loci using inbred mice. Mammalian Genome 20: 437-446

Mackiewicz M, Paigen B, Naidoo N, Pack AI (2008) Analysis of the QTL for sleep homeostasis in mice: Homer1a is a likely candidate. Physiological genomics 33: 91-99

Maret S, Dorsaz S, Gurcel L, Pradervand S, Petit B, Pfister C, Hagenbuchle O, O'Hara BF, Franken P, Tafti M (2007) Homer1a is a core brain molecular correlate of sleep loss. Proceedings of the National Academy of Sciences of the United States of America 104: 20090-20095

Reardon S (2017) Lab mice's ancestral 'Eve' gets her genome sequenced. Nature 551: 281

Shifman S, Bell JT, Copley RR, Taylor MS, Williams RW, Mott R, Flint J (2006) A high-resolution single nucleotide polymorphism genetic map of the mouse genome. PLoS biology 4

Shin D-LL, Pandey AK, Ziebarth JD, Mulligan MK, Williams RW, Geffers R, Hatesuer B, Schughart K, Wilk E (2014) Segregation of a spontaneous Klrd1 (CD94) mutation in DBA/2 mouse substrains. G3 (Bethesda, Md) 5: 235-239
